# Financial Assistance Policy, Hospital Charity Care, and Medical Debt in Collections

**DOI:** 10.1001/jamanetworkopen.2025.55698

**Published:** 2026-01-27

**Authors:** Tatiane Santos, Richard C. Lindrooth, Gary J. Young, Shoou-Yih Lee

**Affiliations:** 1 Celia Scott Weatherhead School of Public Health and Tropical Medicine, Tulane University, New Orleans, Louisiana; 2 Colorado School of Public Health, Aurora; 3 Center for Health Policy and Healthcare Research, Northeastern University, Boston, Massachusetts; 4 D’Amore-McKim School of Business, Northeastern University, Boston, Massachusetts; 5 Bouve College of Health Sciences, Northeastern University, Boston, Massachusetts; 6 Virginia Commonwealth University College of Health Professions, Richmond

## Abstract

**Importance:**

Medical debt is a public health crisis. Hospital bills represent the majority of medical debt and present an opportunity for policy intervention. State financial assistance policies (FAPs) require hospitals to expand patient eligibility. However, it is unknown whether these policies alleviate medical debt.

**Objective:**

To estimate the association of Oregon’s FAP with medical debt in collections and hospitals’ charity care (ie, free or discounted care for patients qualified for FAPs) and bad debt (ie, total amount of uncollectible patient accounts) expenditures.

**Design, Setting, and Participants:**

This cohort study examined county- and hospital-level difference-in-differences and event study models covering the period from 2015 to 2022. This study included 689 counties and 582 hospitals located in Oregon and states that expanded Medicaid in 2014 but did not implement any FAPs. Data were analyzed from January to July 2025.

**Exposure:**

Oregon FAP.

**Main Outcomes and Measures:**

Percentage of the county population with medical debt in collections, and hospital charity care and bad debt expenditures as a percentage of operating expenses.

**Results:**

The sample included 540 hospitals in the control group (43 teaching [7.9%], 199 critical access [36.8%], and 279 metropolitan hospitals [51.6%]) and 42 hospitals in Oregon (3 teaching [7.1%], 15 critical access [35.7%], and 27 metropolitan hospitals [64.3%]) as well as 656 counties in the control group and 33 counties in Oregon. Relative to control states, Oregon’s FAP was associated with a larger decrease in the percentage of the county population with medical debt in collections (−1.67% [95% CI, −3.26% to −0.10%]; *P* = .04), equivalent to 872 to 1180 fewer individuals with medical debt in collections per county. Oregon’s FAP was also associated with larger charity care expenditures (0.31% [95% CI, 0.16% to 0.45%]; *P* = .001), or $227 200 to $639 000 per hospital, compared with the control states. There were no changes in bad debt expenditures.

**Conclusions and Relevance:**

In this cohort study, Oregon’s FAP was associated with a larger decrease in the percentage of the population with medical debt in collections and larger hospital charity care expenditures. State efforts like that of Oregon’s have the potential to alleviate medical debt, especially in light of federal policy changes that are likely to exacerbate the medical debt crisis.

## Key Points

**Question:**

Was Oregon’s financial assistance policy associated with a lower percentage of the population with medical debt in collections and increased hospital charity care spending?

**Findings:**

In this cohort study of 582 hospitals and 689 counties, Oregon’s financial assistance policy was associated with a larger decrease in the percentage of the population with medical debt in collections in the first 3 years after implementation (relative to comparison states). The policy was also associated with larger charity care spending during the same period.

**Meaning:**

These findings suggest that Oregon’s financial assistance policy was associated with reductions in medical debt.

## Teaser

This cohort study examines whether a state-wide financial assistance policy in Oregon is associated with proportion of residents with medical debt in collections and hospital expenditures on charity care and bad debt.

## Introduction

Medical debt is an economic and public health crisis affecting the uninsured and a growing number of commercially insured with high-deductible insurance plans.^1,2^ Depending on how medical debt is defined, approximately 8% to 41% of adults have medical debt, causing financial hardship, including lower credit scores and bankruptcy, and increasing the risks of food insecurity, poor health, and all-cause mortality.^2,3,4^

States have adopted a range of medical debt protection policies to address the growing medical debt crisis.^5^ Upstream policies, such as financial assistance policies (FAPs), aim to prevent debt from occurring, while downstream policies, including limits on credit reporting, protect individuals who already owe medical bills.^5^ In 2023, the 3 major credit bureaus stopped reporting medical debt of $500 or less.^6^ However, this change may offer limited benefit to adults with hospital-related debt, whose balances typically exceed this threshold.^7^

FAPs are considered to hold great potential in alleviating medical debt. While at the federal level, Section 501(r)(4) of the Internal Revenue Service (IRS) requires tax-exempt hospitals to have a written FAP, it does not require hospitals to provide a certain level of financial assistance (FA) to patients based on defined eligibility criteria.^8^ To achieve stronger financial protections for patients, states have implemented their own FAP that require hospitals to set specific eligibility criteria and provide additional protections from extraordinary collections actions (ECA).^5^

Oregon stands out among states as having one of the most patient-friendly FAPs combined with stronger oversight and accountability measures to ensure hospital compliance. Oregon’s FAP, implemented in 2019 under the broader House Bill (HB) 3076, extends financial assistance to insured and uninsured individuals with household income below 400% of the federal poverty level (FPL).^9^ Hospitals are prohibited from sending unpaid bills to collection agencies if they have not screened the patient for financial assistance. Importantly, Oregon’s policy is anchored in a hospital reporting program designed to improve accountability and transparency and to ensure that hospitals adhere to the FAP.^10,11^

Although a study^12^ has examined the short-term impact of Oregon’s FAP on charity care and bad debt, no studies have examined the policy’s impact on medical debt in collections. To fill the gap, our study estimated the association of Oregon’s FAP with medical debt in collections, hospital charity care, and bad debt.

## Methods

We estimated difference-in-differences and event study models to examine whether Oregon’s FAP was associated with reduced medical debt in the local population. A combination of hospital- and county-level analyses enabled us to examine the policy mechanism (ie, hospital charity care and bad debt) and policy impact (ie, medical debt in collections). We hypothesized that Oregon’s FAP would decrease the percentage of the population with medical debt in collections by increasing hospital charity care spending and decreasing hospital bad debt costs.

The study protocol was approved by the Tulane University’s institutional review board. Informed consent was not required because this study did not involve human participants. We followed the Strengthening the Reporting of Observational Studies in Epidemiology (STROBE) reporting guideline.^13^

### Data and Sample

County-level data on medical debt in collections between 2015 to 2022 were sourced from the Urban Institute Credit Bureau Panel.^14^ This is a deidentified, nationally representative sample of consumer data drawn from a major credit bureau.^14^ Data on hospital charity care and bad debt costs were from the RAND Hospital Data, a processed version of the Centers for Medicare & Medicaid Services Healthcare Cost Report Information System.^15^ We merged county-level 2015 to 2022 data from County Health Rankings and Roadmaps and Census Small Area Health Insurance Estimates using county Federal Information Processing System (FIPS) codes.

The control groups for both county- and hospital-level analyses included states that had not implemented FAPs by 2022 and had expanded Medicaid in 2014. The hospital sample was limited to not-for-profit hospitals because Oregon’s FAP does not apply to investor-owned hospitals. The analyses included observations that had outcome data every year from 2015 to 2022, and the hospital sample was winsorized at the first and 99th percentiles of charity care spending to minimize the impact of outliers. After these exclusions, the samples comprised 656 counties (5248 county-years) in the control group and 33 counties in Oregon (264 county-years), and 540 hospitals (4320 hospital-years) in the control group and 42 hospitals (336 hospital-years) in Oregon (eFigure 1 in Supplement 1).

### Primary Outcomes

The primary outcome for the county-level analysis was the percentage of county population with medical debt in collections. Medical debt in collections refers to adults with a credit bureau record that includes medical debt in collections. We examined the median amount of medical debt in collections as a secondary outcome.

The primary outcomes for the hospital-level analysis were charity care and bad debt, both as a percentage of operating expenses. Charity care is defined as free or discounted care provided to patients who qualify for the hospital’s FAP, for which no or partial payment is expected, and no collection efforts are pursued. Bad debt refers to the amount owed by patients for services that the hospital has determined is unlikely to be collected. Once an unpaid bill is deemed uncollectible, hospitals can report it to a credit bureau or sell it to a collections agency after 120 days.^8,9^

### Key Policy: The Evolution of the Oregon FAP

Hospitals in Oregon first became aware that a FAP was under consideration when HB2115 was introduced in 2017.^16^ This bill would have required hospitals to spend at least 5% of operating expenses on community benefits (CB), including charity care.^16^ In early 2018, the Oregon Association of Hospitals and Health Systems helped draft HB4084, another FAP whose provisions were largely later included in HB3076.^17,18,19^ Given this legislative history and the involvement of the Oregon Association of Hospitals and Health Systems, we cannot rule out anticipatory changes in charity care spending. Furthermore, it is possible that hospitals preempted more stringent financial assistance regulations while the provisions in HB3076 were being drafted.^18,19,20^ HB3076 was implemented in 2019 and applies to all services provided by not-for-profit hospitals and their affiliated clinics.^9^ Under the policy, insured and uninsured individuals with household income below 200% FPL qualify for 100% coverage of their out-of-pocket costs, and individuals falling between 200% to 300%, 300% to 350%, and 350% to 400% FPL qualify for a 75%, 50%, and 25% reduction of their out-of-pocket costs, respectively.^9^ Additionally, hospitals cannot charge interest on the medical debt of patients who qualify for financial assistance.^9^ Furthermore, hospitals are required to report yearly charity care expenditures and the number of patient accounts referred for collections and other ECA (eMethods 1 in Supplement 1).^21^

### Statistical Analyses

We estimated linear county- and hospital-level difference-in-differences models to measure the change in the study outcomes following implementation of Oregon’s FAP. Both county- and hospital-level models adjusted for county median income, hospital market concentration as measured by the Herfindahl Hirschman Index, and the percentage of the county population that was uninsured, unemployed, had college education, and nonelderly with a disability. Additionally, hospital-level models adjusted for bed size and teaching status. The models controlled for year fixed effects. County-level models controlled for county fixed effects while hospital-level models controlled for hospital fixed effects. Standard errors were clustered at the state-level. We also conducted event studies to evaluate the trends before and after Oregon’s FAP was implemented. These models included the same covariates as the primary analysis except for the inclusion of Oregon-specific dummy variables that indicated the number of years relative to the policy implementation. Event studies were also used to examine differences in the pre- and postpolicy trends in the outcomes of the treatment vs the control group.

Our specifications for the county-level outcome included a prepolicy period (2015 to 2018) and postpolicy period (2019 to 2022). Specifications for the hospital-level outcomes included four periods that were chosen to measure potential anticipatory effects of the more stringent policies. We defined 2015 to 2016 as the preperiod before any policy was introduced (ie, preanticipatory period) and 2017 to 2018 as the anticipatory period during which HB2115 and HB4084 were being considered; 2019 to 2022 is main the postpolicy period after HB3076 became law; and 2020 to 2022 is the postpolicy preemption period (eMethods 2 and 3 in Supplement 1).

Finally, all county-level models were weighted using county population size to ensure that counties with larger population size had a proportionally greater impact on the estimates while preserving consistency and efficiency in parameter estimates. We performed subanalyses to examine whether the estimates varied by metropolitan status (ie, metropolitan defined as rural-urban continuum codes 1 to 3, and nonmetropolitan otherwise) and terciles of baseline (2018) percentage of the population 400% FPL or less. On average, nonmetropolitan hospitals fare worse financially and may be less able to offer financial assistance to patients.^22^ Additionally, higher rates of uninsurance, underinsurance, and unemployment in rural areas can impact both charity care and bad debt expenditures. Oregon’s FAP was applicable to individuals 400% FPL or less and we’d expect the policy’s impact to be larger in counties that had a higher baseline share of the target population.

Oregon’s FAP was implemented during the same year that COVID-19 emerged. To assess whether COVID-19 fiscal shocks violated the parallel trends and common shocks assumptions we reestimated the hospital-level models with modified total margins as the outcome, calculated by subtracting all expenses reflected in the study’s primary outcomes (ie, charity care, bad debt, and operating expenses). This measure of profitability is an important determinant of charity care spending that takes into account overall financial performance during the public health emergency (PHE) but excludes the financial impact of the policy by design.^23,24^ If the impact of COVID-19 and PHE funding differentially affected the treatment or control group we would expect the postperiod coefficients to be significantly different from zero. In addition, we stratified hospitals by terciles of PHE relief funds as a percentage of operating expenses averaged across 2020 to 2022 to examine whether COVID-19–related financial shocks differentially impacted treatment and control group hospitals.

Additional sensitivity analyses included: (1) unweighted county-level models; (2) inclusion of all Medicaid expansion states regardless of expansion year; and (3) hospital-level analyses limited to hospitals in counties included in the county-level sample (eTables 1 to 4 in Supplement 1). Two-sided tests were considered significant at *P* < .05. Stata version 18.0 (StataCorp) was used to analyze data. Data were analyzed from January to July 2025.

## Results

The sample included 540 hospitals in the control group (43 teaching [7.9%], 199 critical access [36.8%], and 279 metropolitan hospitals [51.6%]) and 42 hospitals in Oregon (3 teaching [7.1%], 15 critical access [35.7%], and 27 metropolitan hospitals [64.3%]) as well as 656 counties in the control group and 33 counties in Oregon. Table 1 presents the descriptive statistics for the hospital-level analysis. Descriptive statistics for the county-level analysis can be found in eTable 5 in Supplement 1. All county and hospital characteristics were reasonably well-balanced between treatment and control groups.

**Table 1.** County and Hospital Baseline Characteristics of Treatment and Control Groups^a^

Characteristics	Mean (SD)		*P* value
	Control group (n = 540)	Oregon (n = 42)	
**Hospital characteristics**			
Charity care as % of operating expenses	1.2 (1.1)	1.8 (0.7)	NA
Bad debt as % of operating expenses	4.9 (3.7)	2.1 (1.7)	NA
Teaching, No. (%)	43 (7.9)	3 (7.1)	.38
Critical access, No. (%)	199 (36.8)	15 (35.7)	.89
Bed size, tercile, No. (%)			
First	201 (37.2)	15 (35.7)	.85
Second	152 (28.1)	17 (40.5)	.10
Third	188 (34.8)	10 (23.8)	.15
Herfindahl-Hirschman Index	0.2 (0.12)	0.2 (0.2)	.79
Operating margins, median (SD), %	3.6 (16.6)	3.9 (11.2)	.58
**County characteristics**			
Population size	373 844 (825 656)	268 688 (261 485)	.41
Median income, $	54 000 (12 090)	56 000 (10 230)	.28
% Uninsured	6.5 (2.4)	7.7 (1.1)	.10
% Unemployed	5.0 (1.7)	5.2 (0.8)	.35
% With some college	63.0 (10.1)	63.9 (9.0)	.56
Metropolitan, No. (%)	279 (51.6)	27 (64.3)	.11
Social vulnerability metric^b^	−0.1 (0.6)	−0.1 (0.6)	.22
Gini Index^c^	0.4 (0.03)	0.4 (0.02)	.59
% Nonelderly with disability	12.7 (4.7)	13.4 (3.4)	.31

Abbreviation: NA, not available.

^a^ County and hospital characteristics are based on prepolicy data (2018). The sample included all not-for-profit hospitals that had complete data from 2015 to 2022 (Oregon, n = 42 or 336 hospital-years; and control, n = 540 or 4320 hospital-years). The *P* value indicates the difference between Oregon and control (hospitals and counties) using Student *t* tests for continuous and χ^2^ tests for binary variables.

^b^ A negative social vulnerability metric indicates that a county is less socially vulnerable, while a positive social vulnerability metric indicates that a county is more socially vulnerable.

^c^ Larger values of the Gini index indicate greater inequality, or a larger gap between the rich and the poor.

**Figure 1.**
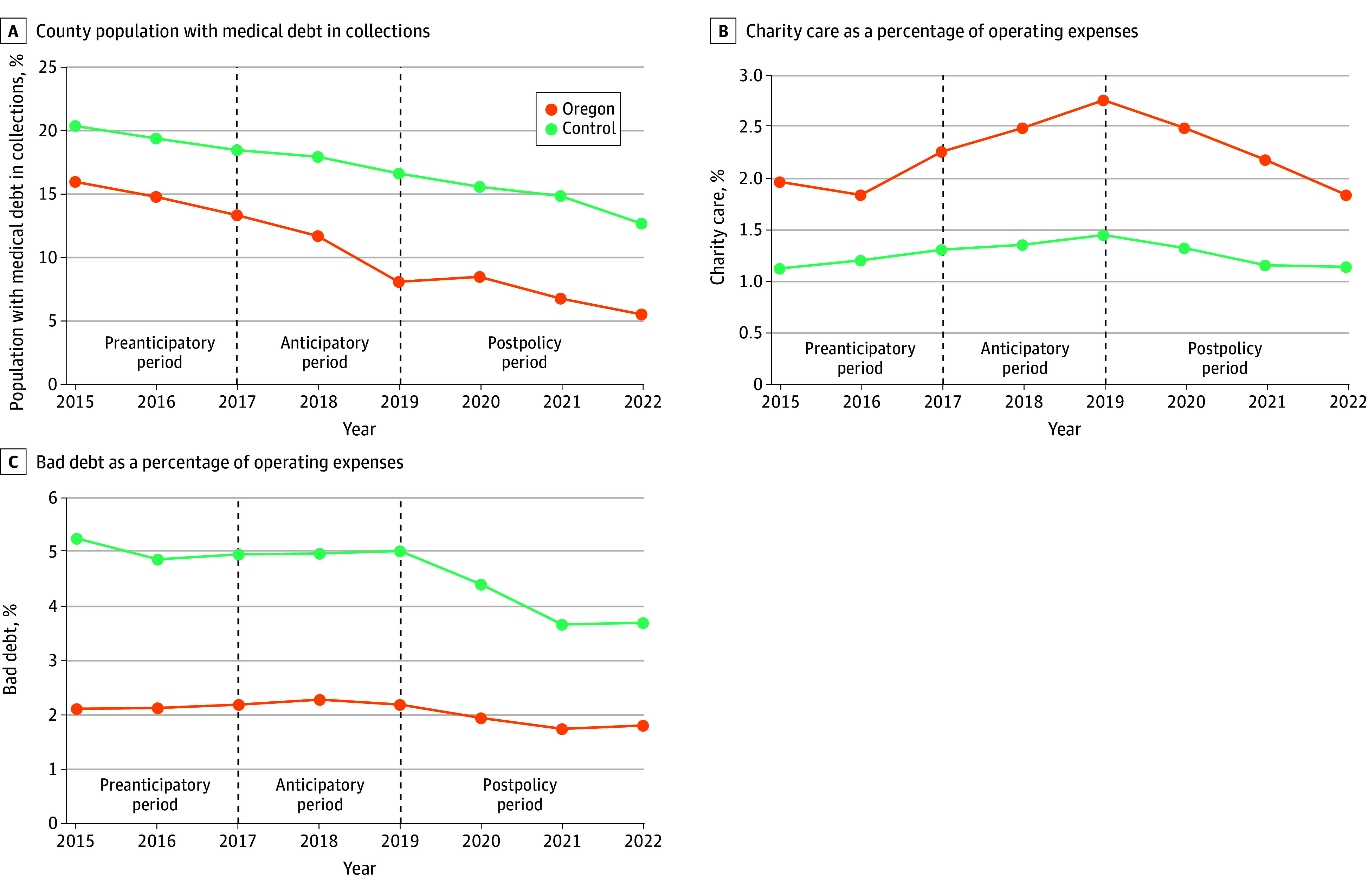
Unadjusted Trends by Treatment Group: Primary Study Outcomes, 2015-2022

We also examined the unadjusted trends of the primary outcomes. Overall, the percentage of the county population with medical debt in collection decreased during the study period. The treatment and control group trends were similar, although the treatment group experienced a larger decrease in the percentage of the population with medical debt in collections from 2015 to 2022 (Figure 1A). Unadjusted charity care expenditures by Oregon hospitals were relatively flat from 2015 to 2016, but trended upwards in 2017 to 2019 during the anticipatory period. The upward trend was reversed after the less stringent FAP (ie, HB3076) was implemented. Charity care spending by control hospitals was steady during the same time period (Figure 1B). Unadjusted trends of bad debt expenditures were similar across treatment and control groups from 2015 to 2019 (Figure 1C). From 2019 to 2021, control hospitals experienced a larger decrease in bad debt expenditures compared with Oregon hospitals, but trends were similar from 2021 to 2022.

**Figure 2.**
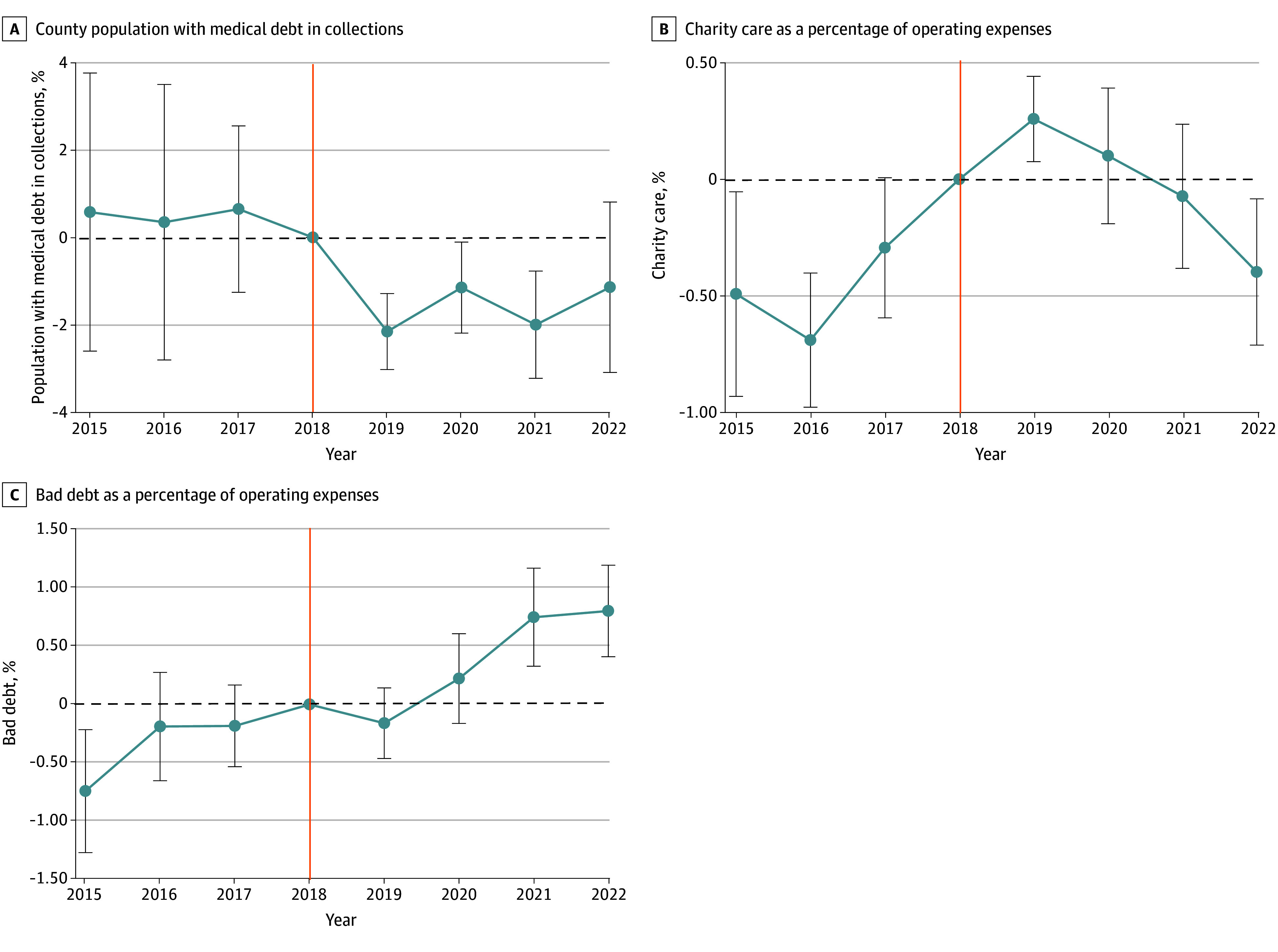
Event Study Difference-in-Differences of County Medical Debt in Collections and Hospital Charity Care and Bad Debt Expenditures Between Treatment and Control Groups, 2015-2022 Whiskers indicate the 95% CIs calculated using standard errors clustered at the state level. Vertical orange line indicates the year before policy implementation.

Table 2 reports the full-sample results of the county-level difference-in-differences models. Oregon’s FAP was associated with a larger decrease in the percentage of the county population with medical debt in collections in the first 3 years postimplementation (2019 to 2022 pooled postpolicy period: −1.67% [95% CI, −3.26% to −0.10%]; *P* = .04; 2019: −2.27% [95% CI, −3.58% to −1.00%]; *P* = .003; 2020: −1.67% [95% CI, −2.91% to −0.44%]; *P* = .01; 2021: −2.00% [95% CI, −3.64% to −0.35%]; *P* = .02). This is equivalent to approximately 872 to 1180 fewer individuals with medical debt in collections per county based on the median population size of Oregon counties. In 2022, there was no statistically significant association. A similar pattern was observed when the sample was stratified by metropolitan status and baseline percentage of the population 400% FPL or less (eTables 6 and 7 and eFigures 2 and 3 in Supplement 1). In secondary analyses, we found that Oregon’s FAP was associated with a lower median amount of medical debt in collections from 2019 to 2021; however, these changes were not sustained in 2022 (eTable 8 in Supplement 1). The event study showed that prepolicy trends were parallel between the treatment and control groups (Figure 2A).

**Table 2.** Association of Oregon Financial Assistance Policy With Percentage of County Population With Medical Debt in Collections, 2015-2022^a^

Outcome	County population with medical debt in collections, mean (SD), %				Unadjusted DID (95% CI), %	Adjusted DID (95% CI), %^b^^,^^c^
	Control group (n = 5248 county-years)		Treatment group (n = 264 county-years)			
	Prepolicy	Postpolicy	Prepolicy	Postpolicy		
Pooled postpolicy period	19.13 (10.05)	14.41 (8.24)	14.00 (4.69)	7.20 (4.18)	−2.08 (−3.02 to −0.26)	−1.67 (−3.26 to −0.10)^d^
Year-specific estimates						
2019	19.13 (10.05)	16.72 (8.74)	14.00 (4.69)	8.11 (4.69)	−3.48 (−3.72 to −1.24)	−2.27 (−3.58 to −1.00)^d^
2020	19.13 (10.05)	15.61 (8.34)	14.00 (4.69)	8.53 (3.99)	−1.95 (−2.58 to −0.16)	−1.67 (−2.91 to −0.44)^d^
2021	19.13 (10.05)	14.92 (8.18)	14.00 (4.69)	6.83 (3.74)	−2.96 (−3.80 to −0.70)	−2.00 (−3.64 to −0.35)^d^
2022	19.13 (10.05)	12.73 (7.12)	14.00 (4.69)	5.62 (3.74)	−1.98 (−3.00 to 1.90)	−0.62 (−3.27 to 2.03)

Abbreviation: DID, difference-in-differences.

^a^ All county-level models were weighted based on each county’s population size. The sample included all counties that had complete data from 2015 to 2022 (Oregon, n = 33 or 264 county-years; and control, n = 656 or 5248 county-years).

^b^ Displays the coefficient from the difference-in-differences estimate using ordinary least squares regression adjusted for control variables (county median income, hospital market concentration as measured by the Herfindahl Hirschman Index, and the percentage of the county population that was uninsured, unemployed, had some college education, and nonelderly with a disability). Models include county and calendar year fixed effects.

^c^ 95% CIs are calculated using standard errors clustered at the state level.

^d^ *P* < .05.

Table 3 reports the full-sample results of the hospital-level difference-in-differences main model and models that examined the potential anticipatory effects. In the main specification (ie, prepolicy period: 2015 to 2018 and postpolicy period: 2019 to 2022), we found that Oregon’s FAP was associated with larger charity care expenditures as a percentage of operating expenses (0.31% [95% CI, 0.16% to 0.45%]; *P* < .001) compared with the control group. This is equivalent to $235 720 to $637 580 per hospital based on median operating expenses of Oregon hospitals. There was no statistically significant association for bad debt in the postpolicy period. The results from the specifications that examined potential anticipatory effects support findings from the main specification, except for the analysis that compared the anticipatory period (2017 to 2018) to the postpolicy preemption period (2020 to 2022) which found no statistically significant association of Oregon’s FAP with charity care (eTables 9 to 13 in Supplement 1). Findings were similar for metropolitan and nonmetropolitan areas (eTable 14 and eFigure 4 in Supplement 1).

**Table 3.** Association of Oregon Financial Assistance Policy With Hospital Charity Care and Bad Debt Expenditures as Percentage of Operating Expenses, 2015-2022

Outcome	Expenditures, mean (SD), % of operating margin				Unadjusted DID (95% CI), %	Adjusted DID (95% CI), %^a^^,^^b^
	Control group (n = 4320 hospital-years)^c^		Treatment group (n = 336 hospital-years)			
	Prepolicy	Postpolicy	Prepolicy	Postpolicy		
Pooled 2015-2018 preperiod vs 2019-2022 postpolicy period						
Charity	1.26 (1.16)	1.27 (1.09)	2.13 (1.03)	2.34 (1.17)	0.20 (0.06 to 0.32)	0.31 (0.16 to 0.45)^d^
Bad debt	5.18 (3.79)	4.34 (3.27)	2.40 (3.85)	1.74 (1.14)	0.18 (−0.15 to 0.26)	0.30 (−0.29 to 0.88)
**Four period estimates**						
2015-2016 preanticipatory period vs 2017-2018 anticipatory period^e^						
Charity	1.18 (1.15)	1.33 (1.16)	1.88 (0.94)	2.38 (1.06)	0.35 (0.14 to 0.52)	0.50 (0.34 to 0.66)^d^
Bad debt	5.18 (3.62)	5.18 (3.96)	2.08 (1.62)	2.70 (5.19)	0.62 (−0.42 to 0.89)	0.33 (−0.75 to 1.41)
2015-2016 preanticipatory period vs 2019-2022 postpolicy period^f^						
Charity	1.18 (1.15)	1.27 (1.09)	1.88 (0.94)	2.34 (1.17)	0.37 (0.19 to 0.56)	0.56 (0.38 to 0.73)^d^
Bad debt	5.18 (3.62)	4.34 (3.27)	2.08 (1.62)	1.74 (1.14)	0.50 (−0.14 to 1.47)	0.81 (−0.11 to 1.73)
2017-2018 anticipatory period vs 2019-2022 postpolicy period^g^						
Charity	1.33 (1.16)	1.27 (1.09)	2.38 (1.06)	2.34 (1.17)	0.02 (−0.03 to 0.10)	0.14 (0.01 to 0.26)^d^
Bad debt	5.18 (3.96)	4.34 (3.27)	2.70 (5.19)	1.74 (1.14)	−0.12 (−0.27 to 0.74)	−0.03 (−0.43 to 0.36)
2017-2018 anticipatory period vs 2020-2022 postpolicy preemption period^h^						
Charity	1.33 (1.16)	1.21 (1.03)	2.38 (1.06)	2.18 (1.03)	−0.08 (−0.14 to 0.17)	0.04 (−0.10 to 0.18)
Bad debt	5.18 (3.96)	4.05 (3.00)	2.70 (5.19)	1.68 (1.20)	0.71 (−0.98 to 1.25)	0.23 (−0.35 to 0.81)

Abbreviation: DID, difference-in-differences; HB, House Bill.

^a^ Displays the coefficient from the difference-in-differences estimate using ordinary least squares regression adjusted for control variables (hospital bed size, teaching status, county median income, hospital market concentration as measured by the Herfindahl Hirschman Index, and the percentage of the county population that was uninsured, unemployed, had some college education, and nonelderly with a disability). Models include hospital and calendar year fixed effects.

^b^ 95% CIs are calculated using standard errors clustered at the state level.

^c^ The sample included all not-for-profit hospitals that had complete data from 2015 to 2022 (Oregon, n = 42 or 336 hospital-years; and control, n = 540 or 4320 hospital-years).

^d^ *P* < .05.

^e^ Compares anticipatory period (ie, when HB2115 and HB4084 were introduced but failed) to the pre-anticipatory period (ie, when no bills were introduced and there was no overt effort to reform financial assistance policies).

^f^ Compares the postpolicy period (ie, when HB3076 was passed and implemented) to the preanticipatory period.

^g^ Compares the anticipatory period to the postpolicy period.

^h^ This comparison examines whether hospitals preempted more stringent financial assistance regulations while the provisions contained in HB3076 were being drafted.

The event study suggests that the parallel trends assumption was violated for charity care expenditures (Figure 2B). This was likely due to anticipatory behavior by hospitals. Indeed, setting the reference period to 2015 (ie, preanticipatory period) eliminated the pretrend. For more details on the anticipatory behavior by hospitals, refer to eFigure 6 in Supplement 1. The event study for bad debt expenditures shows that prepolicy trends were parallel between the treatment and control groups and that Oregon hospitals incurred larger bad debt expenditures in 2021 and 2022 compared with control hospitals. (Figure 2C).

The stratified analyses by PHE relief funds (eTable 15 in Supplement 1) show a positive and statistically significant association across all terciles for charity care, indicating that the main model results are not driven by hospitals with greater COVID-19–related financial support. We found no statistically significant association for bad debt in the analyses stratified by PHE relief funds. Finally, the analysis that used the modified total margin as the outcome shows that COVID-19 financial impacts are balanced in the treatment and control groups (eFigure 7 in Supplement 1). This further supports the main model findings and indicates that Oregon’s FAP had broad impact independent of COVID-19–related financial shocks.

## Discussion

To our knowledge, our study is the first to examine the association of a state-level FAP with medical debt in collections in parallel with the policy’s association with hospital charity care and bad debt expenditures. Oregon’s FAP applied to nearly all hospitals in the state (58 of 60 hospitals) and was expected to affect most patients with household incomes below 400% FPL. Overall, we found that, relative to the comparison states, Oregon’s FAP was associated with a larger decrease in medical debt in collections. The decrease amounted to approximately 872 to 1180 fewer individuals per Oregon county with medical debt in collections. The county-level changes were supported by the finding that Oregon hospitals incurred larger charity care expenditures, approximately $235 720 to $637 580 per hospital, from 2019 to 2021. There is some evidence of an attenuation in the policy’s impact starting in 2022, both in terms of medical debt in collections and hospital charity care spending. Our hospital-level findings were aligned with a previous study that found larger charity care expenditures relative to the control group.^12^

We found some evidence of an anticipatory response by hospitals due to legislative attempts to reform financial assistance in Oregon predating HB3076.^16,17^ The Oregon Association of Hospitals and Health Systems was involved in these early efforts; therefore, it is reasonable to expect that hospitals may have adjusted their FAP ahead of HB3076’s passage.^18,19^ We found evidence that after HB3076 passed, with its less strict financial assistance requirements, hospital charity care spending began to revert to lower levels consistent with the implemented FAP.

Importantly, Oregon’s FAP is a component of the broader regulatory effort in HB3076, designed to improve hospitals’ accountability, transparency, and investment in their communities. Another key component of the bill is the CB minimum spending floor implemented in 2021. The spending floor formula is based on hospitals’ revenues, financial health, and prior levels of unreimbursed care spending.^25^ Each hospital is assigned a minimum dollar amount that they must spend on areas including charity care, community health improvement, and other CB spending categories.^9^ Our findings raise the possibility that the 2 phases of HB3076 may be in conflict with each other. Phase 1 (ie, FAP) led Oregon hospitals to incur larger charity care expenditures immediately after implementation. However, phase 2 (ie, spending floor) may have attenuated the effectiveness of the FAP as hospitals allocated fewer dollars to charity care and may have spent more on other categories of CB. It’s also possible that the minimum spending floors were set lower than what hospitals would have spent absent the policy, and that hospitals responded by decreasing their CB spending overall. A similar phenomenon, dubbed race to the bottom, has been observed with Texas’ charity care spending floor.^26^

We observed no immediate change in hospital bad debt expenditures following Oregon’s FAP; however, event studies showed higher reported bad debt in 2021 to 2022 relative to controls. Before implementation, hospitals waited 120 days before referring unpaid bills to collections. Under HB3076, hospitals were prohibited from referring unpaid bills to collections before screening patients for financial assistance. To demonstrate compliance, hospitals may have wanted to document multiple attempts to contact patients about applying for assistance, further extending the time before referral to collections.^27^ If hospitals recognize bad debt only after unpaid bills are referred to collections, as is common, these requirements could delay the timing of bad debt accounting in financial reports. A potential consequence is that, as hospitals refer older unpaid bills to collections, Oregon could experience an increase in residents with medical debt in collections. To prevent this, policymakers could consider protections such as Colorado’s 2023 ban on reporting medical debt, although a recent CFPB interpretive rule may limit state authority to do so.^28,29^

There are opportunities to refine Oregon’s, and other states’, FAPs. In fact, the Oregon Health Authority (OHA) has already amended HB3076 to improve transparency and accountability.^30^ Starting in 2024, hospitals must screen patients for presumptive eligibility for financial assistance.^30^ In 2025, hospitals were required to report data on the number of financial assistance applications approved and denied, median per person debt, and total number of accounts referred to a collection agency.^30^ Future policy amendments can be tailored to protect financially vulnerable hospitals, especially rural hospitals that are at high risk of closure.^31^ Upcoming Medicaid funding cuts are projected to reduce hospital revenues by over $31 billion and increase their uncompensated care costs by over $6 billion.^32^ Oregon’s spending floors already account for the financial ability of hospitals to spend on charity care and CB. The spending floor formula can be updated to account for the shifting federal policy landscape and protect financially vulnerable hospitals.

### Limitations

The study has limitations. First, our measure of medical debt in collections did not capture medicals bills that are past due but not yet sent to collections, paid with a credit card or paid through payment plans with the practice or hospital, or debt owed to a family member or friend. Our estimates were likely conservative, representing the lower bound of the policy effect, as they captured a smaller portion of total medical debt. Second, we could not observe the specific actions taken by hospitals on bad debt. Third, because the policy period overlapped with the COVID-19 pandemic, pandemic-related financial shocks may have influenced outcomes. However, results were consistent across terciles of PHE relief funds, and a model using modified total margin as the outcome showed no association with the policy, suggesting the main findings are unlikely driven by pandemic-related financial shocks.

## Conclusions

In this cohort study, we found that Oregon’s FAP was associated with a larger decrease in the percentage of the population with medical debt in collections and larger hospital charity care expenditures in the first 3 years after implementation. Our findings suggest that Oregon’s FAP and the broader HB3076 have the potential to alleviate medical debt, especially in the context of federal policy changes that appear likely to exacerbate the medical debt crisis. In July 2025, a federal judge removed a CFPB rule that would have removed $49 billion in medical debt from the credit reports of millions of Americans.^33^ The Congressional Budget Office has estimated that approximately 14 million individuals will become uninsured by 2034 under the Congress-approved budget reconciliation package that cut Medicaid funding and made changes to Affordable Care Act marketplaces.^34^ Oregon’s existing regulatory framework provides a fertile ground for continued refinements that can help the state achieve its goal of alleviating medical debt and increasing hospital investment in their communities. Importantly, other states can learn from the Oregon experiment and avoid some of the early implementation challenges elucidated by our study.

## Notes

Supplement 1. **eFigure 1.** CONSORT Diagram for County and Hospital Samples **eFigure 2.** Event Study Analysis of Medical Debt in Collections, 2015-2022, by Metropolitan Status **eFigure 3.** Event Study Analysis of Medical Debt in Collections, 2015-2022, by Tercile of Baseline Percentage of Population 400% FPL or Less **eFigure 4.** Event Study Analysis of Hospital Charity Care and Bad Debt Expenditures By Metropolitan Status, 2015-2022 **eFigure 5.** Event Study Analysis of Hospital Charity Care and Bad Debt Expenditures By Tercile of Baseline Percentage of Population 400% FPL or Less, 2015-2022 **eFigure 6.** Event Study Analysis of Hospital Charity Care for the Full Sample and By Metropolitan Status, 2015-2022; Reference Period Set to 2015 **eFigure 7. **Robustness Check to COVID-19 Fiscal Shocks: Event Study Analysis Using Modified Total Margin as the Outcome **eMethods 1.** Description of Oregon Financial Assistance Policy and Oregon House Bill 3076 **eMethods 2.** Two-Way Fixed Effects Difference-In-Differences Model Specification; Oregon Financial Assistance Policy: County **eMethods 3. **Two-Way Fixed Effects Difference-In-Differences Model Specification; Oregon Financial Assistance Policy: Hospital **eTable 1.** Full Estimates Output (County)—Association of Oregon Financial Assistance Policy with Percentage of County Population With Medical Debt in Collections, 2015-2022; Full Sample Without Population Weighting **eTable 2.** Full Estimates Output (County)—Association of Oregon Financial Assistance Policy with Percentage of County Population With Medical Debt in Collections, 2015-2022; Sample Includes All Medicaid Expansion States **eTable 3.** Full Estimates Output (Hospital)—Association of Oregon Financial Assistance Policy With Charity Care and Bad Debt, 2015-2022; Sample Includes All Medicaid Expansion States **eTable 4.** Full Estimates Output (Hospital)—Association of Oregon Financial Assistance Policy With Charity Care and Bad Debt, 2015-2022; Sample Limited to Counties Included in County Analyses **eTable 5.** County Baseline Characteristics of Treatment and Control Groups: County Analysis **eTable 6.** Full Estimates Output (County)—Association of Oregon Financial Assistance Policy with Percentage of County Population With Medical Debt in Collections, 2015-2022; Full Sample and By Metropolitan Status **eTable 7.** Full Estimates Output (County)—Association of Oregon Financial Assistance Policy with Percent of County Population With Medical Debt in Collections, 2015-2022; By Tercile of Baseline Percentage of Population 400% FPL or Less **eTable 8.** Full Estimates Output (County)—Association of Oregon Financial Assistance Policy With Median Amount of Medical Debt in Collections, 2015-2022, Full Sample **eTable 9.** Main Specification: Pooled 2015-2018 Prepolicy Period vs 2019-2022 Postpolicy Period Full Estimates Output (Hospital)—Association of Oregon Financial Assistance Policy With Charity Care and Bad Debt, 2015-2022 **eTable 10.** Anticipatory Effect Model: 2015-2016 Preanticipatory Period vs 2017-2018 Anticipatory Period; Full Estimates Output (Hospital)—Association of Oregon Financial Assistance Policy With Charity Care and Bad Debt, 2015-2022 **eTable 11.** Anticipatory Effect Model: 2015-2016 Pre-Anticipatory Period vs 2019-2022 Postpolicy Period; Full Estimates Output (Hospital)—Association of Oregon Financial Assistance Policy With Charity Care and Bad Debt, 2015-2022 **eTable 12.** Anticipatory Effect Model: 2017-2018 Anticipatory Period vs 2019-2022 Postpolicy Period; Full Estimates Output (Hospital)—Association of Oregon Financial Assistance Policy With Charity Care and Bad Debt, 2015-2022 **eTable 13.** Anticipatory Effect Model: 2017-2018 Anticipatory Period vs 2020-2022 Postpolicy Preemption Period; Full Estimates Output (Hospital)—Association of Oregon Financial Assistance Policy With Charity Care and Bad Debt, 2015-2022 **eTable 14.** Full Estimates Output (Hospital)—Association of Oregon Financial Assistance Policy With Charity Care and Bad Debt, 2015-2022, Full Sample and By Metropolitan Status **eTable 15.** Robustness Check to COVID-19 Fiscal Shocks: Full Estimates Output—Association of Oregon Financial Assistance Policy With Charity Care and Bad Debt, 2015-2022 By Tercile of Public Health Emergency Relief Funds

Supplement 2.
Data Sharing Statement
