# Supplementary material for: Financial Assistance Policy, Hospital Charity Care, and Medical Debt in Collections
Source: JAMA Netw Open. 2026 Jan 27;9(1):e2555698. doi: 10.1001/jamanetworkopen.2025.55698 (PMC12848624; doi:10.1001/jamanetworkopen.2025.55698)
Supplement: Supplement 1. — eFigure 1. CONSORT Diagram for County and Hospital Samples eFigure 2. Event Study Analysis of Medical Debt in Collections, 2015-2022, by Metropolitan Status eFigure 3. Event Study Analysis of Medical Debt in Collections, 2015-2022, by Tercile of Baseline Percentage of Population 400% FPL or Less eFigure 4. Event Study Analysis of Hospital Charity Care and Bad Debt Expenditures By Metropolitan Status, 2015-2022 eFigure 5. Event Study Analysis of Hospital Charity Care and Bad Debt Expenditures By Tercile of Baseline Percentage of Population 400% FPL or Less, 2015-2022 eFigure 6. Event Study Analysis of Hospital Charity Care for the Full Sample and By Metropolitan Status, 2015-2022; Reference Period Set to 2015 eFigure 7. Robustness Check to COVID-19 Fiscal Shocks: Event Study Analysis Using Modified Total Margin as the Outcome eMethods 1. Description of Oregon Financial Assistance Policy and Oregon House Bill 3076 eMethods 2. Two-Way Fixed Effects Difference-In-Differences Model Specification; Oregon Financial Assistance Policy: County eMethods 3. Two-Way Fixed Effects Difference-In-Differences Model Specification; Oregon Financial Assistance Policy: Hospital eTable 1. Full Estimates Output (County)—Association of Oregon Financial Assistance Policy with Percentage of County Population With Medical Debt in Collections, 2015-2022; Full Sample Without Population Weighting eTable 2. Full Estimates Output (County)—Association of Oregon Financial Assistance Policy with Percentage of County Population With Medical Debt in Collections, 2015-2022; Sample Includes All Medicaid Expansion States eTable 3. Full Estimates Output (Hospital)—Association of Oregon Financial Assistance Policy With Charity Care and Bad Debt, 2015-2022; Sample Includes All Medicaid Expansion States eTable 4. Full Estimates Output (Hospital)—Association of Oregon Financial Assistance Policy With Charity Care and Bad Debt, 2015-2022; Sample Limited to Counties Included in County Analyses eTable 5. County B [file jamanetwopen-e2555698-s001.pdf]

Supplemental Online Content

Santos T, Lindrooth RC, Young GJ, Lee SY. Financial assistance policy, hospital charity care, and medical debt in collections. *JAMA Netw Open*. 2026;9(1):e2555698. doi:10.1001/jamanetworkopen.2025.55698

eFigure 1. CONSORT Diagram for County and Hospital Samples

eFigure 2. Event Study Analysis of Medical Debt in Collections, 2015-2022; By Metropolitan Status

eFigure 3. Event Study Analysis of Medical Debt in Collections, 2015-2022; By Tercile of Baseline Percentage of Population 400% FPL or Less

eFigure 4. Event Study Analysis of Hospital Charity Care and Bad Debt Expenditures By Metropolitan Status, 2015-2022

eFigure 5. Event Study Analysis of Hospital Charity Care and Bad Debt Expenditures By Tercile of Baseline Percentage of Population 400% FPL or Less, 2015-2022

eFigure 6. Event Study Analysis of Hospital Charity Care for the Full Sample and By Metropolitan Status, 2015-2022; Reference Period Set to 2015

eFigure 7. Robustness Check to COVID-19 Fiscal Shocks: Event Study Analysis Using Modified Total Margin as the Outcome

eMethods 1. Description of Oregon Financial Assistance Policy and Oregon House Bill 3076

eMethods 2. Two-Way Fixed Effects Difference-In-Differences Model Specification; Oregon Financial Assistance Policy: County

eMethods 3. Two-Way Fixed Effects Difference-In-Differences Model Specification; Oregon Financial Assistance Policy: Hospital

eTable 1. Full Estimates Output (County)—Association of Oregon Financial Assistance Policy with Percentage of County Population With Medical Debt in Collections, 2015-2022; Full Sample Without Population Weighting

eTable 2. Full Estimates Output (County)—Association of Oregon Financial Assistance Policy with Percentage of County Population With Medical Debt in Collections, 2015-2022; Sample Includes All Medicaid Expansion States

eTable 3. Full Estimates Output (Hospital)—Association of Oregon Financial Assistance Policy With Charity Care and Bad Debt, 2015-2022; Sample Includes All Medicaid Expansion States

eTable 4. Full Estimates Output (Hospital)—Association of Oregon Financial Assistance Policy With Charity Care and Bad Debt, 2015-2022; Sample Limited to Counties Included in County Analyses

eTable 5. County Baseline Characteristics of Treatment and Control Groups: County Analysis

© 2026 Santos T et al. *JAMA Network Open*.

eTable 6. Full Estimates Output (County)—Association of Oregon Financial Assistance Policy with Percentage of County Population With Medical Debt in Collections, 2015-2022; Full Sample and By Metropolitan Status

eTable 7. Full Estimates Output (County)—Association of Oregon Financial Assistance Policy with Percentage of County Population With Medical Debt in Collections, 2015-2022; By Tercile of Baseline Percentage of Population 400% FPL or Less

eTable 8. Full Estimates Output (County)—Association of Oregon Financial Assistance Policy With Median Amount of Medical Debt in Collections, 2015-2022; Full Sample

eTable 9. Main Specification: Pooled 2015-2018 Prepolicy Period vs 2019-2022 Postpolicy Period Full Estimates Output (Hospital)—Association of Oregon Financial Assistance Policy With Charity Care and Bad Debt, 2015-2022

eTable 10. Anticipatory Effect Model: 2015-2016 Preanticipatory Period vs 2017-2018 Anticipatory Period; Full Estimates Output (Hospital)—Association of Oregon Financial Assistance Policy With Charity Care and Bad Debt, 2015-2022

eTable 11. Anticipatory Effect Model: 2015-2016 Pre-Anticipatory Period vs 2019-2022 Postpolicy Period; Full Estimates Output (Hospital)—Association of Oregon Financial Assistance Policy With Charity Care and Bad Debt, 2015-2022

eTable 12. Anticipatory Effect Model: 2017-2018 Anticipatory Period vs 2019-2022 Post-Policy Period; Full Estimates Output (Hospital)—Association of Oregon Financial Assistance Policy With Charity Care and Bad Debt, 2015-2022

eTable 13. Anticipatory Effect Model: 2017-2018 Anticipatory Period vs 2020-2022 Postpolicy Preemption Period; Full Estimates Output (Hospital)—Association of Oregon Financial Assistance Policy With Charity Care and Bad Debt, 2015-2022

eTable 14. Full Estimates Output (Hospital)—Association of Oregon Financial Assistance Policy With Charity Care and Bad Debt, 2015-2022, Full Sample and By Metropolitan Status

eTable 15. Robustness Check to COVID-19 Fiscal Shocks: Full Estimates Output—Association of Oregon Financial Assistance Policy With Charity Care and Bad Debt, 2015-2022 By Tercile of Public Health Emergency Relief Funds

This supplemental material has been provided by the authors to give readers additional information about their work.

© 2026 Santos T et al. *JAMA Network Open*.

eFigure 1 in Supplement 1. CONSORT Diagram for County and Hospital Samples

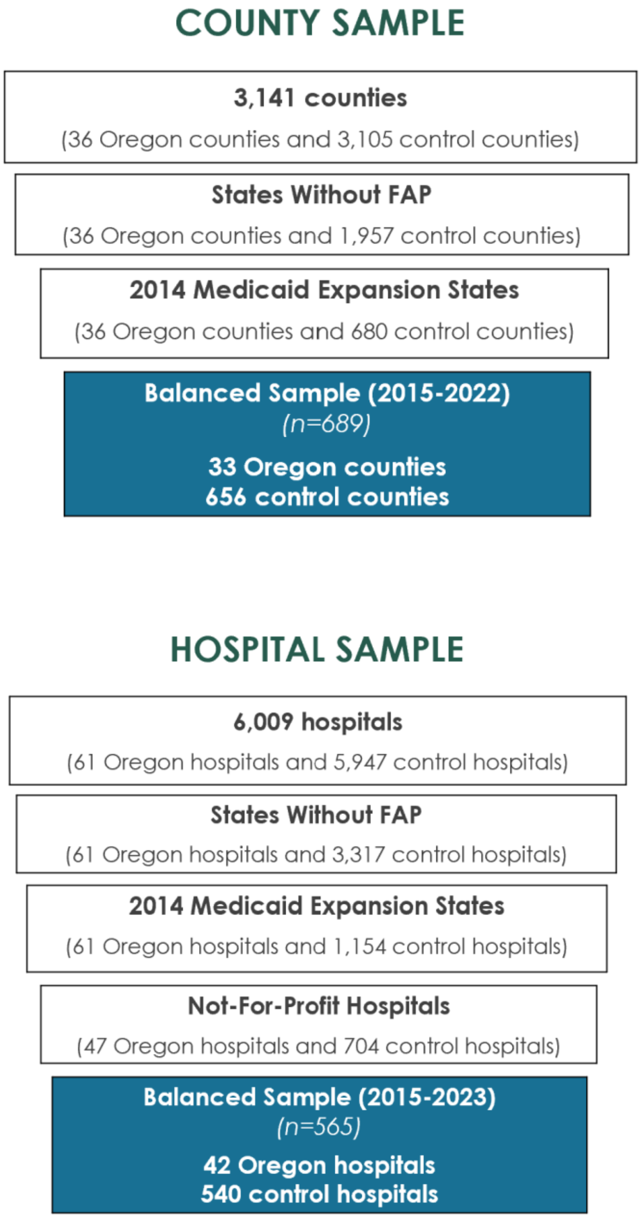

Note: Only counties and hospitals that had data every single year during the study period (2015-2022) were included (i.e., balanced sample).

- The Urban Institute Changing Medical Debt Landscape in the United States database has some missing values because Changing Medical Debt Landscape in the United States.
  - 21 unique counties were dropped from the sample due to missing data in at least 1 year from 2015 to 2022.
  - The Oregon counties excluded were: Gilliam, Sherman, and Wheeler.
- 160 unique hospitals were dropped from the sample due to missing data in at least 1 year from 2015 to 2022.

© 2026 Santos T et al. *JAMA Network Open.*

eMethods 1. Description of Oregon Financial Assistance Policy and Oregon House Bill 3076

Note: Here we provide information on key components of Oregon's financial assistance policy and House Bill (HB3076). For more details, please refer to House Bill 3076.
https://olis.oregonlegislature.gov/liz/2019R1/Downloads/MeasureDocument/HB3076/Enrolled

HB3076 was signed into law on 6/25/2019. All medical debt protections were effective upon passage and applied to charges occurring in 2019 after the signing of the bill.

The medical debt provisions are found in Section 4 of the bill (see HB3076 for more details). It requires hospitals to screen patients for financial assistance (FA) prior to taking extraordinary collection actions (e.g., sending unpaid bills to a debt collector or collections) and to make their financial assistance applications widely available. Additionally, hospitals may not charge interest on the medical debt of patients who qualify for financial assistance. If a patient does not qualify for FA then the hospital or other debt collector may only charge interest on the patient's medical debt starting the week before the date when the patient was billed, at a specified, standardized rate: between 2-5% per annum as determined by the Board of Governors of the Federal Reserve System. Hospitals and debt collectors cannot collect debt from a patient's child or other family members that are not financially responsible for the debt. Debt cannot be collected in any way that violates these laws.

Importantly, Section 4 of HB3076 was made part of Oregon's fair debt collection laws, and as such violations become unlawful collection practice under ORS 646.639. This allows a patient a private right of action (i.e., they can sue) if a hospital violates these provisions.

Section 2 of HB3076 articulates the FA eligibility tiers and additional details about the FAP. Under Section 2, insured and uninsured individuals with household income below 200% FPL qualify for 100% coverage of their out-of-pocket costs, and individuals falling between 200-300%, 300-350%, and 350-400% FPL qualify for a 75%, 50%, and 25% reduction of their out-of-pocket costs, respectively. The FAP applies to all of the not-for-profit hospitals including all their affiliated clinics. Additionally, hospitals must proactively screen patients for presumptive eligibility for financial assistance, translate application materials, and provide interpreting services for patients whose first language is not English.

Notably, there are no direct civil penalties associated with the spending floor. According the a representative from the Oregon Health Authority (OHA), the consequences are two fold:

1- Public reporting and accountability. Media picks up and reports when OHA publishes spending floors and hospitals must speak to it. "Hospitals generally like to avoid negative press, so this has proven to be a strong motivator."
2- The Department of Justice (DOJ) monitors the spending floor and uses it as part of the charitable programs oversight.

There is likely no direct consequential outcome in missing a spending floor in any single year. There would be consequences and DOJ follow up if a hospital missed multiple spending floors. Furthermore, hospitals generally don't have any chance to rectify their non-compliance other than to improve the next year and get in compliance."

© 2026 Santos T et al. *JAMA Network Open.*

Note on Anticipatory Behavior by Oregon Hospitals:

It is possible that Oregon hospitals adjusted their financial assistance policies in anticipation of HB3076 being implemented. The study's first author (Dr. Santos) has maintained close contact with representatives from the Oregon Health Authority (OHA) who lead all efforts related to HB3076. Additionally, Dr. Santos has spoken to the original authors of the bill; that is, Service Employees International Union (SEIU) Local 49. SEIU representatives who were involved with drafting the language for HB3076 confirmed that the Oregon Association of Hospitals and Health Systems were key partners in these early efforts which began as early as 2018. Due to the hospital association's close involvement with drafting HB3076; it is reasonable to expect an anticipatory effect; that is, hospitals adjusted their financial assistance policies ahead of HB3076's passage.

There were other legislative attempts to reform financial assistance policy in Oregon which predate HB3076. In 2017, HB2115 (see link below for details on the bill), a stricter regulation would have set a minimum community benefit standard of 5% of hospitals' gross receipts (i.e., charity care is part of community benefit). However, HB2115 did not make it out of committee. In early 2018, HB4084 (see link below for details on the bill) contained a lot of the same provisions as HB3076, and indeed, several provisions from HB4084 were worked into HB3076. Notably, we found several news articles (see links below) that suggest that the Oregon Association of Hospitals and Health System was closely monitoring and lobbying against the earlier efforts.

1- **HB2115:** https://olis.oregonlegislature.gov/liz/2017R1/Downloads/MeasureDocument/HB2115/Introduced
2- **HB4084:** https://legiscan.com/OR/bill/HB4084/2018
3- **Media article about HB4084:** https://www.thelundreport.org/content/hospital-association-kills-greenlicks-attempt-clarify-charity-care-patients
4- **Media article on HB4084:** Oregon hospitals to clarify financial aid policies - KTVZ
5- **Media article on legislation for expanded financial assistance by hospitals:** https://www.thelundreport.org/content/oregon-legislators-could-limit-hospital-tax-breaks-increase-financial-scrutiny

© 2026 Santos T et al. *JAMA Network Open*.

eMethods 2 in Supplement 1. Two-Way Fixed Effects Difference-In-Differences Model Specification; Oregon Financial Assistance Policy: County

The model for percent of the county population with medical debt in collections is:

$$\boldsymbol{Y_{ct}} = \alpha + \beta_1\ Oregon_c \times Post\ FAP_{t=2019-2022} + \ \phi\mathrm{x}_{\mathrm{ct}} + \lambda_{\mathrm{c}} + \gamma_{\mathrm{t}} + \ \varepsilon_{ct}$$

where '*c*' denotes county and 't' denotes calendar year. Y_{ct} is the percentage of the county population with medical debt in collections (i.e., primary outcome) for county *'c'* in calendar year *'t'*. $Oregon_c$ is a dummy treatment indicator where 1 represents a county located in Oregon and 0 otherwise. $Post\ FAP_{t=2019-2023}$ is a dummy variable that represents the period after policy implementation (i.e., 2019) and equals 1 for calendar years 2019 to 2022. $\phi\mathrm{x}_{\mathrm{ct}}$ is a vector of county-level covariates including median income, hospital market concentration as measured by the Herfindahl Hirschman Index (i.e. defined as the sum of all hospitals' squared market shares within each respective market), and the percent of the county population that was uninsured, unemployed, had some college education, and non-elderly with a disability. λ_{c} is a county fixed effect and γ_{t} is a calendar year fixed effect. The coefficient β_1 measures the average change in the primary outcome in Oregon counties relative to the change experienced in the control group, after implementation of Oregon's financial assistance policy. We also estimated models that included a dummy variable for each year after policy implementation. In these models, there were 4 separate difference-in-differences estimators, β_1 to β_4 for 2019, 2020, 2021, and 2022 respectively. ε_{ct} represents the error term. Standard errors clustered at the state-level.

We also conducted an event study to evaluate the trends before and after Oregon's financial assistance policy was implemented. These models included the same covariates as equation 1 except for the inclusion of Oregon-specific dummy variables that indicated the number of years relative to the implementation of Oregon's financial assistance policy. The event studies were also used to test whether the pre-policy trends were parallel between treatment and control groups.

© 2026 Santos T et al. *JAMA Network Open.*

eMethods 3 in Supplement 1. Two-Way Fixed Effects Difference-In-Differences Model Specification; Oregon Financial Assistance Policy: Hospital

The model for hospital charity care and bad debt expenditure (as a percent of operating expenses) is:

$$\boldsymbol{Y_{ht}} = \alpha + \beta_1 Oregon_h \times Post\ FAP_{t=2019-2022} + \delta \mathrm{x_{ht}} + \phi \mathrm{x_{ct}} + \lambda_{\mathrm{h}} + \gamma_{\mathrm{t}} + \varepsilon_{hct} \quad (1)$$

where '*h*' denotes hospital and 't' denotes calendar year. $\mathrm{Y_{ct}}$ is the primary outcome (i.e., charity care and bad debt as a percentage of operating expenses) for hospital *'h'* in calendar year *'t'*. $Oregon_h$ is a dummy treatment indicator where 1 represents a hospital located in Oregon and 0 otherwise. $Post\ FAP_{t=2019-2023}$ is a dummy variable that represents the period after policy implementation (i.e., 2019) and equals 1 for calendar years 2019 to 2022. $\delta \mathrm{x_{ht}}$ is a vector of hospital covariates including bed size and teaching status. $\phi \mathrm{x_{ct}}$ is a vector of county-level covariates including median income, hospital market concentration as measured by the Herfindahl Hirschman Index (i.e. defined as the sum of all hospitals' squared market shares within each respective market), and the percent of the county population that was uninsured, unemployed, had some college education, and non-elderly with a disability. λ_{h} is a hospital fixed effect and γ_{t} is a calendar year fixed effect. The coefficient β_1 measures the average change in the primary outcomes by Oregon hospitals relative to the change experienced in the control group, after implementation of Oregon's financial assistance policy. We also estimated models that included a dummy variable for each year after policy implementation. In these models, there were 4 separate difference-in-differences estimators, β_1 to β_4 for 2019, 2020, 2021, and 2022 respectively ε_{ct} represents the error term. Standard errors clustered at the state-level.

We also conducted an event study to evaluate the trends before and after Oregon's financial assistance policy was implemented. These models included the same covariates as equation 1 except for the inclusion of Oregon-specific dummy variables that indicated the number of years relative to the implementation of Oregon's financial assistance policy. Event studies were used to examine differences in the pre- and post-policy trends in the outcomes of the treatment versus the control group

Anticipatory Effect Models

Our specifications for the hospital-level outcomes included three-periods that were chosen to measure potential anticipatory effects of the more stringent policies. We defined: 2015-2016 as the pre-period before any policy was introduced (i.e., pre-anticipatory period); 2017-2018 as the anticipatory period during which HB 2115 and HB4084 were being considered; and 2019-2022 is the post-policy period after HB3076 became law and the FAP was implemented. Specifically, we estimated the main specification (model 1) using the following pre- and post-periods.

1- Pooled 2015-2018 Pre-Period vs. 2019-2022 Post-Policy Period (main model)
2- 2015-2016 pre-period vs 2017-2018 Anticipatory Period
3- 2015-2016 pre-period vs. 2019-2022 Post-Policy Period
4- 2017-2018 Anticipatory Period vs. 2019-2022 Post-Policy Period
5- 2017-2018 Anticipatory Period vs. 2020-2022 Post-Policy Period

© 2026 Santos T et al. *JAMA Network Open.*

eTable 1. Full Estimates Output (County) - Association of Oregon Financial Assistance Policy with Percent of County Population With Medical Debt in Collections, 2015-22; Full Sample WITHOUT POPULATION WEIGHTING

	**FULL SAMPLE**
	n=264 county-years (Treatment)<br>n=5,248 county-years (Control)
**DID (2019 post)** [a]	-0.0257*
	(0.0120)
**DID (2020 post)**	-0.0156
	(0.0111)
**DID (2021 post)**	-0.0241*
	(0.0120)
**DID (2022 post)**	-0.0153
	(0.0151)
Median Income (per $10,000s)	4.10e-07
	(2.74e-07)
Uninsured %	0.440
	(0.291)
Unemployment %	0.226
	(0.241)
Some College %	0.0201
	(0.0355)
HHI [b]	-3.47e-06*
	(1.85e-06)
Non-elderly with disability %	0.0607
	(0.0497)
2016	-0.00757
	(0.00430)
2017	0.00401
	(0.0141)
2018	0.00880
	(0.0163)
2019	0.000636
	(0.0173)
2020	-0.0109
	(0.0177)
2021	-0.0197

© 2026 Santos T et al. *JAMA Network Open.*

	(0.0178)
2022	-0.0502**
	(0.0163)
Constant	0.0780*
	(0.0406)

Abbreviations: DID, difference-in-differences

[a] Displays the coefficient from the difference-in-differences estimate using ordinary least squares regression adjusted for control variables (county median income, hospital market concentration as measured by the Herfindahl Hirschman Index, and the percent of the county population that was uninsured, unemployed, had some college education, and non-elderly with a disability). Models include county and calendar year fixed effects. Standard errors clustered at the state level. *** p<0.01, ** p<0.05, * p<0.1

[b] In the county models, the Herfindahl Hirschman Index (HHI) is calculated by squaring the market share (based on adjusted admissions) of each unique health system and independent hospital competing within each hospital referral region and then summing the resulting numbers. The sample was restricted to hospitals providing general medical and surgical services and exclude federal hospitals. To create the HHI measure at the county level for each year, each HRR was cross-walked to a county and used population weights from the Geocor 2022.

eTable 2. Full Estimates Output (County) - Association of Oregon Financial Assistance Policy with Percent of County Population With Medical Debt in Collections, 2015-22; Sample Includes All Medicaid Expansion States

	**FULL SAMPLE**
	n=264 county-years (Treatment) n=10,000 county-years (Control)
**DID (2019 post)** [a]	-0.0281***
	(0.00450)
**DID (2020 post)**	-0.0258***
	(0.00470)
**DID (2021 post)**	-0.0286***
	(0.00529)
**DID (2022 post)**	-0.0158*
	(0.00786)
Median Income (per $10,000s)	5.23e-08
	(3.68e-07)
Uninsured %	0.379**
	(0.155)
Unemployment %	0.182
	(0.119)
Some College %	-0.0355
	(0.0242)
HHI [b]	-2.34e-06
	(3.49e-06)
Non-elderly with disability %	0.0486
	(0.0842)
2016	-0.00583***
	(0.00203)
2017	-0.00186
	(0.00601)
2018	0.00227
	(0.00864)
2019	-0.00199
	(0.00971)
2020	-0.00862
	(0.00975)
2021	-0.0196*

© 2026 Santos T et al. *JAMA Network Open.*

	(0.0102)
2022	-0.0525***
	(0.0105)
Constant	0.136***
	(0.0465)

Abbreviations: DID, difference-in-differences

[a] Displays the coefficient from the difference-in-differences estimate using ordinary least squares regression adjusted for control variables (county median income, hospital market concentration as measured by the Herfindahl Hirschman Index, and the percent of the county population that was uninsured, unemployed, had some college education, and non-elderly with a disability). Models include county and calendar year fixed effects. Standard errors clustered at the state level. *** p<0.01, ** p<0.05, * p<0.1

[b] In the county models, the Herfindahl Hirschman Index (HHI) is calculated by squaring the market share (based on adjusted admissions) of each unique health system and independent hospital competing within each hospital referral region and then summing the resulting numbers. The sample was restricted to hospitals providing general medical and surgical services and exclude federal hospitals. To create the HHI measure at the county level for each year, each HRR was cross-walked to a county and used population weights from the Geocor 2022.

eTable 3. Full Estimates Output (Hospital) - Association of Oregon Financial Assistance Policy with Charity Care and Bad Debt, 2015-22; Sample Includes All Medicaid Expansion States

	**Charity Care**	**Bad Debt**
	n=336 hospital-years (Treatment)<br>n=7,591 hospital-years (Control)	
**DID (2019 post)** [a]	0.00743***	-0.00116
	(0.00103)	(0.00241)
**DID (2020 post)**	0.00611***	0.00374
	(0.00126)	(0.00259)
**DID (2021 post)**	0.00543***	0.00909***
	(0.00148)	(0.00254)
**DID (2022 post)**	0.00237	0.0124***
	(0.00141)	(0.00277)
2nd Bed Tercile *(ref. 1st Tercile)*	0.00462	-0.000836
	(0.00284)	(0.00650)
3rd Bed Tercile *(ref. 1st Tercile)*	0.00507*	0.00314
	(0.00292)	(0.00577)
Teaching	0.00105	-0.00494
	(0.00134)	(0.00324)
HHI [b]	0.00946	-0.00275
	(0.0132)	(0.0248)
Median Income (per $1,000s)	-0.00234**	0.00111
	(0.000959)	(0.00183)
Uninsured %	0.0518*	0.162***
	(0.0264)	(0.0527)
Unemployment %	-0.0394	0.0271
	(0.0259)	(0.0693)
Some College %	-0.0125	-0.00808
	(0.00908)	(0.0182)
Non-elderly with disability %	0.0323*	-0.125
	(0.0162)	(0.0728)
2016	0.000994	-0.00126
	(0.000815)	(0.00165)
2017	0.00228*	0.00132
	(0.00123)	(0.00318)
2018	0.00336**	0.00242
	(0.00142)	(0.00372)
2019	0.00384**	0.00161
	(0.00169)	(0.00431)
2020	0.00269	-0.00506
	(0.00162)	(0.00413)

© 2026 Santos T et al. *JAMA Network Open.*

2021	0.000195	-0.0121***
	(0.00165)	(0.00389)
2022	-0.000575	-0.0137***
	(0.00182)	(0.00420)
Constant	0.0260**	0.0637***
	(0.0103)	(0.0213)

Abbreviations: DID, difference-in-differences; CI, confidence interval

[a] Displays the coefficient from the difference-in-differences estimate using ordinary least squares regression adjusted for control variables (hospital bed size, teaching status, county median income, hospital market concentration as measured by the Herfindahl Hirschman Index, and the percent of the county population that was uninsured, unemployed, had some college education, and non-elderly with a disability). Models include hospital and calendar year fixed effects.

Standard errors clustered at the state level. *** p<0.01, ** p<0.05, * p<0.1

[b] In the hospital models, Herfindahl Hirschman Index was defined as the sum of all hospitals' squared market shares within each hospital referral region.

eTable 4. Full Estimates Output (Hospital) - Association of Oregon Financial Assistance Policy with Charity Care and Bad Debt, 2015-22; Sample Limited to Counties Included in County Analyses

	**Charity Care**	**Bad Debt**
	n=336 hospital-years (Treatment)<br>n=4,286 hospital-years (Control)	
**DID (2019 post)** [a]	0.00583***	-0.00389
	(0.000817)	(0.00317)
**DID (2020 post)**	0.00418***	0.000982
	(0.000713)	(0.00307)
**DID (2021 post)**	0.00254**	0.00717*
	(0.000841)	(0.00346)
**DID (2022 post)**	-0.000792	0.00834**
	(0.000844)	(0.00309)
2nd Bed Tercile *(ref. 1st Tercile)*	0.00185	-0.00393
	(0.00226)	(0.00647)
3rd Bed Tercile *(ref. 1st Tercile)*	0.000252	0.000985
	(0.00209)	(0.00503)
Teaching	-0.000376	-0.00417
	(0.00175)	(0.00267)
HHI [b]	-0.00926	0.00751
	(0.00879)	(0.0211)
Median Income (per $1,000s)	-0.00103	-0.000851
	(0.000860)	(0.00316)
Uninsured %	0.0413	0.179**
	(0.0271)	(0.0730)
Unemployment %	-0.00465	0.0486
	(0.0182)	(0.0777)
Some College %	-0.00346	0.0247
	(0.00627)	(0.0286)
Non-elderly with disability %	0.0388**	-0.0960**
	(0.0146)	(0.0430)
2016	0.00133	-0.000431
	(0.000836)	(0.00159)
2017	0.00316**	0.00439
	(0.00111)	(0.00427)
2018	0.00448***	0.00656
	(0.00135)	(0.00451)
2019	0.00555***	0.00776
	(0.00167)	(0.00596)
2020	0.00440**	0.00154
	(0.00175)	(0.00511)

© 2026 Santos T et al. *JAMA Network Open.*

2021	0.00266	-0.00644
	(0.00179)	(0.00437)
2022	0.00266	-0.00647
	(0.00195)	(0.00403)
Constant	0.0101	0.0112
	(0.00897)	(0.0169)

Abbreviations: DID, difference-in-differences; CI, confidence interval

[a] Displays the coefficient from the difference-in-differences estimate using ordinary least squares regression adjusted for control variables (hospital bed size, teaching status, county median income, hospital market concentration as measured by the Herfindahl Hirschman Index, and the percent of the county population that was uninsured, unemployed, had some college education, and non-elderly with a disability). Models include hospital and calendar year fixed effects.

Standard errors clustered at the state level. *** p<0.01, ** p<0.05, * p<0.1

[b] In the hospital models, Herfindahl Hirschman Index was defined as the sum of all hospitals' squared market shares within each hospital referral region.

© 2026 Santos T et al. *JAMA Network Open.*

eTable 5. County Baseline Characteristics of Treatment and Control Groups: County Analysis

	**Control Group** *(n=656)*	**Oregon** *(n=33)*	***p-value***
**County Characteristics**			
Medical Debt in Collections (% of population)	18.0	11.7	
Median Medical Debt in Collections ($)	621.0	781.0	
Population Size	74,331	125,388	0.21
Herfindahl-Hirschman Index [a]	2,537	2,354	0.76
Median Income [b]	8.0	8.3	0.26
Uninsurance (%)	7.9	9.4	0.07
Some College (%)	5.8	6.0	0.31
White (%)	90.0	88.5	0.48
Metro (%)	32.3	45.4	0.12
Social Vulnerability Index [c]	0.2	0.2	0.14
Gini Index [d]	0.4	0.4	0.90
Non-elderly with disability (%)	14.4	14.8	0.65

County characteristics are based on pre-policy data (2018). The sample includes all counties that have complete data from 2015 to 2022 (Oregon, n=33 or 264 county-years; and Control, n=656 or 5,248 county-years). The P value indicates the difference between Oregon counties and control counties using Student t tests for continuous and $\chi 2$ tests for binary variables.

[a] In the county models, the Herfindahl Hirschman Index (HHI) is calculated by squaring the market share (based on adjusted admissions) of each unique health system and independent hospital competing within each hospital referral region and then summing the resulting numbers. The sample was restricted to hospitals providing general medical and surgical services and exclude federal hospitals. To create the HHI measure at the county level for each year, each HRR was cross-walked to a county and used population weights from the Geocor 2022.

[b] Median annual household income is standardized by $10000

[c] Larger values of the social vulnerability metric indicate higher levels of social vulnerability.

[d] Larger values of the Gini index indicate greater inequality, or a larger gap between the rich and the poor.

© 2026 Santos T et al. *JAMA Network Open.*

eTable 6. Full Estimates Output (County) - Association of Oregon Financial Assistance Policy with Percent of County Population With Medical Debt in Collections, 2015-22; Full Sample and By Metropolitan Status

	**FULL SAMPLE [a]**	**NON-METROPOLITAN COUNTIES [b]**	**METROPOLITAN COUNTIES [c]**
	n=264 county-years (Treatment) n=5,248 county-years (Control)	n=144 county-years (Treatment) n=3,552 county-years (Control)	n=120 county-years (Treatment) n=1,696 county-years (Control)
**DID (2019 post) [d]**	-0.0227***	-0.0209	-0.0218***
	(0.00586)	(0.0125)	(0.00404)
**DID (2020 post)**	-0.0167**	-0.0156	-0.0154**
	(0.00553)	(0.0117)	(0.00518)
**DID (2021 post)**	-0.0200**	-0.0235*	-0.0174**
	(0.00739)	(0.0126)	(0.00748)
**DID (2022 post)**	-0.00622	-0.0165	-0.00179
	(0.0119)	(0.0153)	(0.0131)
Median Income (per $10,000s)	-2.68e-07	1.65e-07	-6.55e-07
	(5.74e-07)	(5.15e-07)	(7.97e-07)
Uninsured %	0.524**	0.453	0.560**
	(0.227)	(0.311)	(0.189)
Unemployment %	0.192	0.244	0.167
	(0.149)	(0.213)	(0.125)
Some College %	-0.0335	-0.0227	-0.0484
	(0.0335)	(0.0275)	(0.0584)
HHI [e]	3.94e-07	-8.62e-07	4.87e-07
	(3.80e-06)	(3.81e-06)	(4.21e-06)
Non-elderly with disability %	0.0874	0.0905	0.106
	(0.118)	(0.0589)	(0.294)
2016	-0.00648**	-0.00575	-0.00617**
	(0.00247)	(0.00475)	(0.00224)
2017	0.00966	0.00802	0.0116*
	(0.00855)	(0.0161)	(0.00614)
2018	0.0159	0.0114	0.0196*
	(0.0127)	(0.0210)	(0.0100)
2019	0.0105	0.00136	0.0162
	(0.0148)	(0.0220)	(0.0128)
2020	0.000122	-0.00956	0.00666
	(0.0156)	(0.0222)	(0.0142)
2021	-0.0101	-0.0182	-0.00372

	(0.0157)	(0.0217)	(0.0145)
2022	-0.0443**	-0.0520**	-0.0373**
	(0.0167)	(0.0198)	(0.0164)
Constant	0.132*	0.112	0.169
	(0.0691)	(0.0660)	(0.106)

Abbreviations: DID, difference-in-differences

Metropolitan defined as Rural Urban Continuum Code (RUCC) 1-3; Non-metropolitan defined as RUCC 4-9

All county-level models were weighted based on each county's population size.

[a] The full sample included all counties that had complete data from 2015 to 2022 (Oregon, n=33 or 264 county-years; and Control, n=656 or 5,248 county-years).

[b] The sample limited to non-metropolitan counties included all counties that had complete data from 2015 to 2022 (Oregon, n=18 or 144 county-years; and Control, n=444 or 3,552 county-years).

[c] The sample limited to metropolitan counties included all counties that had complete data from 2015 to 2022 (Oregon, n=15 or 120 county-years; and Control, n=212 or 1,696 county-years).

[d] Displays the coefficient from the difference-in-differences estimate using ordinary least squares regression adjusted for control variables (county median income, hospital market concentration as measured by the Herfindahl Hirschman Index, and the percent of the county population that was uninsured, unemployed, had some college education, and non-elderly with a disability). Models include county and calendar year fixed effects. Standard errors clustered at the state level. *** p<0.01, ** p<0.05, * p<0.1

[e] In the county models, the Herfindahl Hirschman Index (HHI) is calculated by squaring the market share (based on adjusted admissions) of each unique health system and independent hospital competing within each hospital referral region and then summing the resulting numbers. The sample was restricted to hospitals providing general medical and surgical services and exclude federal hospitals. To create the HHI measure at the county level for each year, each HRR was cross-walked to a county and used population weights from the Geocor 2022.

© 2026 Santos T et al. *JAMA Network Open.*

eFigure 2. Event Study Analysis of Medical Debt in Collections By Metropolitan Status, 2015-22

Non-Metropolitan Counties [a]

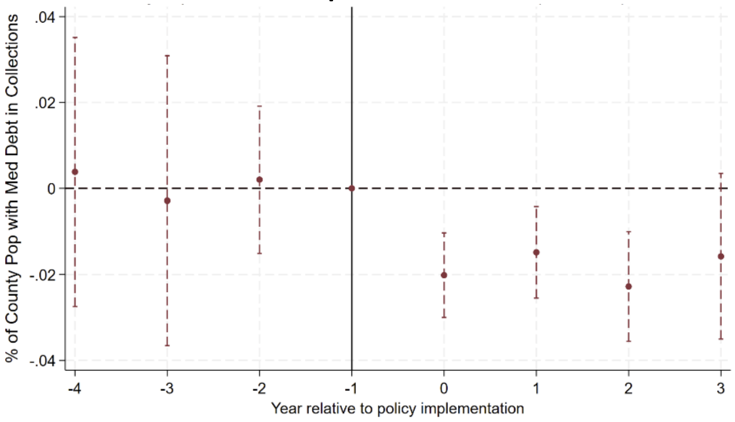

Metropolitan Counties [b]

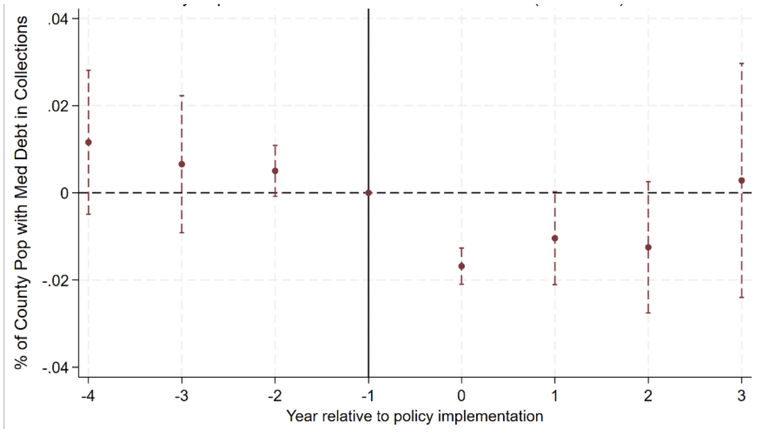

Metropolitan defined as Rural Urban Continuum Code (RUCC) 1-3; Non-metropolitan defined as RUCC 4-9

[a] The sample limited to non-metropolitan counties included all counties that had complete data from 2015 to 2022 (Oregon, n=18 or 144 county-years; and Control, n=444 or 3,552 county-years).

[b] The sample limited to metropolitan counties included all counties that had complete data from 2015 to 2022 (Oregon, n=15 or 120 county-years; and Control, n=212 or 1,696 county-years).

All county-level models were weighted based on each county's population size. Models adjusted for control variables (county median income, hospital market concentration as measured by the Herfindahl Hirschman Index, and the percent of the county population that was uninsured, unemployed, had some college education, and non-elderly with a disability). Models include county and calendar year fixed effects.

95% CIs are calculated using standard errors clustered at the state level. Time at −1, the reference period, indicates the year before implementation of Oregon's financial assistance policy.

eTable 7. Full Estimates Output (County) - Association of Oregon Financial Assistance Policy with Percent of County Population With Medical Debt in Collections, 2015-22; By Tercile of Baseline Percent of Population =<400 FPL

	**1st Tercile**	**2nd Tercile**	**3rd Tercile**
	n=88 county-years (Treatment) n=1,752 county-years (Control)	n=88 county-years (Treatment) n=1,752 county-years (Control)	n=88 county-years (Treatment) n=1,744 county-years (Control)
**DID (2019 post)** [a]	-0.0171**	-0.0433***	-0.0163
	(0.00557)	(0.00501)	(0.0101)
**DID (2020 post)**	-0.0166**	-0.0119	-0.00471
	(0.00592)	(0.00710)	(0.00905)
**DID (2021 post)**	-0.0139	-0.0259**	-0.0198*
	(0.00830)	(0.00995)	(0.00957)
**DID (2022 post)**	-0.00196	-0.0103	-1.77e-06
	(0.0143)	(0.0100)	(0.0116)
Median Income (per $10,000s)	-0.00686	-0.0165***	0.000490
	(0.00903)	(0.00438)	(0.00347)
Uninsured %	0.546**	0.187	0.358
	(0.232)	(0.229)	(0.340)
Unemployment %	0.0284	-0.00994	0.341
	(0.0924)	(0.123)	(0.194)
Some College %	-0.0771	0.0711	-0.0354
	(0.0662)	(0.122)	(0.0337)
HHI [b]	5.80e-07	1.12e-05**	1.50e-06
	(4.78e-06)	(4.79e-06)	(4.44e-06)
Non-elderly with disability %	0.0629	0.138	0.0507
	(0.254)	(0.246)	(0.0736)
2016	-0.00619**	-0.0103**	-0.00807
	(0.00247)	(0.00383)	(0.00703)
2017	0.00834	-0.00670	0.00727
	(0.00770)	(0.00754)	(0.0196)
2018	0.0155	-0.00920	0.00783
	(0.0118)	(0.0143)	(0.0263)
2019	0.0128	-0.0217	-0.00343
	(0.0140)	(0.0186)	(0.0282)
2020	0.00501	-0.0371	-0.0176
	(0.0143)	(0.0205)	(0.0289)
2021	-0.00499	-0.0464*	-0.0252
	(0.0144)	(0.0225)	(0.0286)
2022	-0.0288*	-0.0759***	-0.0721**

© 2026 Santos T et al. *JAMA Network Open.*

	(0.0159)	(0.0220)	(0.0247)
Constant	0.196	0.231**	0.172**
	(0.124)	(0.101)	(0.0629)

Abbreviations: DID, difference-in-differences

All county-level models were weighted based on each county's population size.

Each tercile included counties that had complete data from 2015 to 2022 (Oregon, n=11 or 88 county-years; and Control, n=219 or 1,752 county-years). Except for the third tercile control group which had 218 counties or 1,744 county-years.

[a] Displays the coefficient from the difference-in-differences estimate using ordinary least squares regression adjusted for control variables (county median income, hospital market concentration as measured by the Herfindahl Hirschman Index, and the percent of the county population that was uninsured, unemployed, had some college education, and non-elderly with a disability). Models include county and calendar year fixed effects. Standard errors clustered at the state level. *** p<0.01, ** p<0.05, * p<0.1

[b] In the county models, the Herfindahl Hirschman Index (HHI) is calculated by squaring the market share (based on adjusted admissions) of each unique health system and independent hospital competing within each hospital referral region and then summing the resulting numbers. The sample was restricted to hospitals providing general medical and surgical services and exclude federal hospitals. To create the HHI measure at the county level for each year, each HRR was cross-walked to a county and used population weights from the Geocor 2022.

eFigure 3. Event Study Analysis of Medical Debt in Collections, 2015-22; By Tercile of Baseline Percent of Population =<400 FPL

1st Tercile of % of Baseline Population =<400% FPL [a]

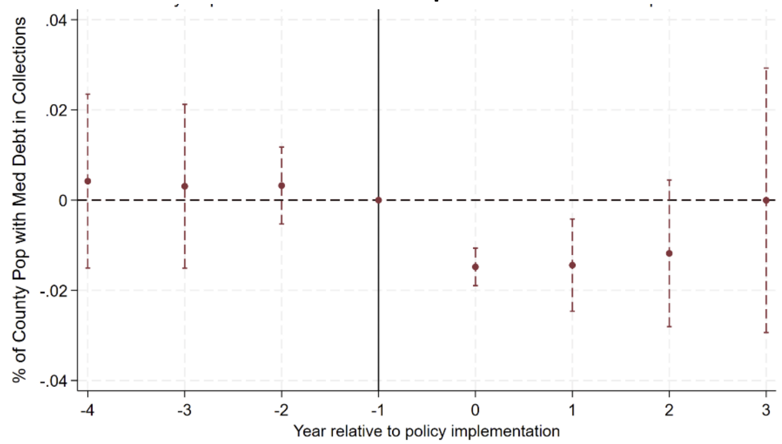

2nd Tercile of % of Baseline Population =<400% FPL

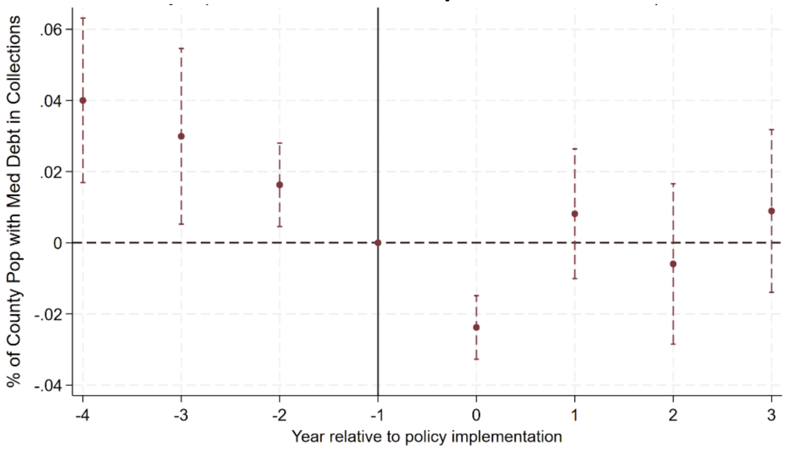

3rd Tercile of % of Baseline Population =<400% FPL

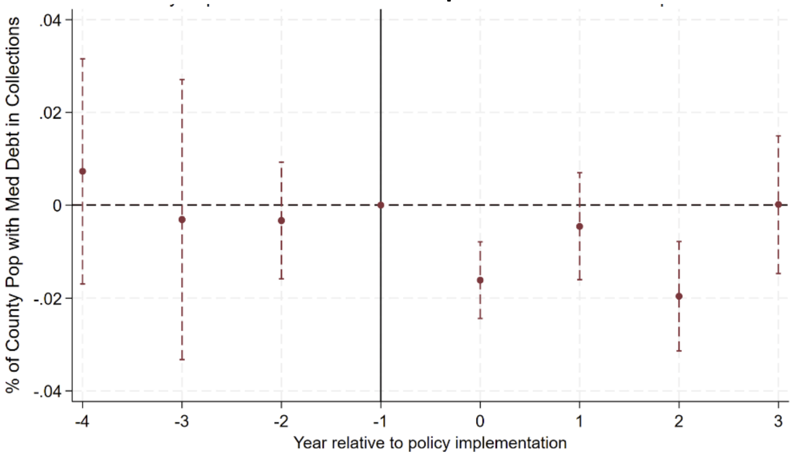

© 2026 Santos T et al. *JAMA Network Open.*

[a] Each tercile included counties that had complete data from 2015 to 2022 (Oregon, n=11 or 88 county-years; and Control, n=219 or 1,752 county-years). Except for the third tercile control group which had 218 counties or 1,744 county-years.

All county-level models were weighted based on each county's population size. Models adjusted for control variables (county median income, hospital market concentration as measured by the Herfindahl Hirschman Index, and the percent of the county population that was uninsured, unemployed, had some college education, and non-elderly with a disability). Models include county and calendar year fixed effects.

95% CIs are calculated using standard errors clustered at the state level. Time at −1, the reference period, indicates the year before implementation of Oregon's financial assistance policy.

© 2026 Santos T et al. *JAMA Network Open.*

eTable 8. Full Estimates Output (County) - Association of Oregon Financial Assistance Policy with Median Amount of Medical Debt in Collections, 2015-22; Full Sample

	**Median Amount of Medical Debt in Collections**
	n=88 county-years (Treatment)<br>n=2,032 county-years (Control)
**DID (2019 post)** [a]	-160.7***
	(14.66)
**DID (2020 post)**	-166.5***
	(25.69)
**DID (2021 post)**	-108.1***
	(33.87)
**DID (2022 post)**	-24.89
	(63.58)
Median Income (per $10,000s)	-0.00525
	(0.00523)
Uninsured %	2,891***
	(810.1)
Unemployment %	1,757**
	(647.2)
Some College %	-132.2
	(243.3)
HHI [b]	0.0426
	(0.0315)
Non-elderly with disability %	-847.1
	(508.3)
2016	1.635
	(16.68)
2017	120.7**
	(50.38)
2018	168.1*
	(82.16)
2019	253.0**
	(101.7)
2020	250.7*
	(114.7)
2021	215.2
	(124.1)
2022	9.166
	(118.1)

© 2026 Santos T et al. *JAMA Network Open.*

Constant	772.1
	(555.4)

Abbreviations: DID, difference-in-differences

All county-level models were weighted based on each county's population size.

The sample included counties that had complete data from 2015 to 2022 (Oregon, n=11 or 88 county-years; and Control, n=254 or 2,032 county-years).

[a] Displays the coefficient from the difference-in-differences estimate using ordinary least squares regression adjusted for control variables (county median income, hospital market concentration as measured by the Herfindahl Hirschman Index, and the percent of the county population that was uninsured, unemployed, had some college education, and non-elderly with a disability). Models include county and calendar year fixed effects. Standard errors clustered at the state level. *** p<0.01, ** p<0.05, * p<0.1

[b] In the county models, the Herfindahl Hirschman Index (HHI) is calculated by squaring the market share (based on adjusted admissions) of each unique health system and independent hospital competing within each hospital referral region and then summing the resulting numbers. The sample was restricted to hospitals providing general medical and surgical services and exclude federal hospitals. To create the HHI measure at the county level for each year, each HRR was cross-walked to a county and used population weights from the Geocor 2022.

© 2026 Santos T et al. *JAMA Network Open.*

eTable 9. Main Specification: Pooled 2015-2018 Pre-Policy Period vs. 2019-2022 Post-Policy Period Full Estimates Output (Hospital) - Association of Oregon Financial Assistance Policy with Charity Care and Bad Debt, 2015-22

	**Charity Care**	**Bad Debt**
	n=336 hospital-years (Treatment)<br>n=4,320 hospital-years (Control)	
**DID (2019-2022 post)**[a]	0.00307***	0.00297
	(0.000635)	(0.00264)
2nd Bed Tercile *(ref. 1st Tercile)*	0.00140	0.00221
	(0.00196)	(0.00448)
3rd Bed Tercile *(ref. 1st Tercile)*	0.000417	0.00668
	(0.00149)	(0.00382)
Teaching	-0.000108	-0.00462
	(0.00183)	(0.00264)
HHI[b]	-0.0138	0.0131
	(0.00816)	(0.0189)
Median Income (per $1,000s)	-0.000877	-0.000800
	(0.000897)	(0.00294)
Uninsured %	0.0429	0.143*
	(0.0269)	(0.0703)
Unemployment %	0.00501	0.0468
	(0.0156)	(0.0701)
Some College %	-0.00542	0.0304
	(0.00546)	(0.0288)
Non-elderly with disability %	0.0313**	-0.0811*
	(0.0117)	(0.0405)
2016	0.00142*	-0.00155
	(0.000756)	(0.00145)
2017	0.00321***	0.00312
	(0.000990)	(0.00398)
2018	0.00475***	0.00486
	(0.00112)	(0.00380)
2019	0.00587***	0.00522
	(0.00142)	(0.00509)
2020	0.00467**	-0.000506
	(0.00148)	(0.00422)
2021	0.00291*	-0.00791*
	(0.00160)	(0.00386)
2022	0.00268	-0.00786**
	(0.00185)	(0.00351)
Constant	0.0163	0.0121
	(0.00941)	(0.0154)

Abbreviations: DID, difference-in-differences; CI, confidence interval

The sample included all not-for-profit hospitals that had complete data from 2015 to 2022 (Oregon, n=42 or 336 hospital-years; and Control, n=540 or 4,320 hospital-years).

[a] Displays the coefficient from the difference-in-differences estimate using ordinary least squares regression adjusted for control variables (hospital bed size, teaching status, county median income, hospital market concentration as measured by the Herfindahl Hirschman Index, and the percent of the county population that was uninsured, unemployed, had some college education, and non-elderly with a disability). Models include hospital and calendar year fixed effects.

[b] In the hospital models, Herfindahl Hirschman Index was defined as the sum of all hospitals' squared market shares within each hospital referral region.

Standard errors clustered at the state level. *** p<0.01, ** p<0.05, * p<0.1

eTable 11. Anticipatory Effect Model: 2015-2016 Pre-Anticipatory Period vs. 2019-2022 Post-Policy Period; Full Estimates Output (Hospital) - Association of Oregon Financial Assistance Policy with Charity Care and Bad Debt, 2015-22

	Charity Care	Bad Debt
	n=252 hospital-years (Treatment)<br>n=3,240 hospital-years (Control)	
**DID (2019-2022 post)** [a]	0.00559***	0.00810*
	(0.000780)	(0.00413)
2nd Bed Tercile *(ref. 1st Tercile)*	0.00228	0.00571
	(0.00221)	(0.00485)
3rd Bed Tercile *(ref. 1st Tercile)*	0.00117	0.0113**
	(0.00191)	(0.00382)
Teaching	0.000137	-0.00586
	(0.00186)	(0.00428)
HHI [b]	-0.0121	0.0227
	(0.00991)	(0.0194)
Median Income (per $1,000s)	-0.00106	-0.00156
	(0.000902)	(0.00317)
Uninsured %	0.0610**	0.229***
	(0.0246)	(0.0714)
Unemployment %	0.00129	-0.00351
	(0.0186)	(0.0968)
Some College %	-0.00465	0.0622*
	(0.00564)	(0.0297)
Non-elderly with disability %	0.0303*	-0.103***
	(0.0138)	(0.0306)
2016	0.00165**	-0.000890
	(0.000737)	(0.00134)
2019	0.00643***	0.00691
	(0.00133)	(0.00520)
2020	0.00524***	0.00110
	(0.00138)	(0.00437)
2021	0.00349**	-0.00622
	(0.00150)	(0.00383)
2022	0.00324*	-0.00630
	(0.00176)	(0.00349)
Constant	0.0172	0.00341
	(0.0109)	(0.0174)

Abbreviations: DID, difference-in-differences; CI, confidence interval

© 2026 Santos T et al. *JAMA Network Open.*

The sample included all not-for-profit hospitals that had complete data from 2015 to 2022 (Oregon, n=42 or 336 hospital-years; and Control, n=540 or 4,320 hospital-years).

[a] Displays the coefficient from the difference-in-differences estimate using ordinary least squares regression adjusted for control variables (hospital bed size, teaching status, county median income, hospital market concentration as measured by the Herfindahl Hirschman Index, and the percent of the county population that was uninsured, unemployed, had some college education, and non-elderly with a disability). Models include hospital and calendar year fixed effects.

[b] In the hospital models, Herfindahl Hirschman Index was defined as the sum of all hospitals' squared market shares within each hospital referral region.

Standard errors clustered at the state level. *** p<0.01, ** p<0.05, * p<0.1

eTable 12. Anticipatory Effect Model: 2017-2018 Anticipatory Period vs. 2019-2022 Post-Policy Period; Full Estimates Output (Hospital) - Association of Oregon Financial Assistance Policy with Charity Care and Bad Debt, 2015-22

	**Charity Care**	**Bad Debt**
	n=252 hospital-years (Treatment)<br>n=3,240 hospital-years (Control)	
**DID (2019-2022 post)** [a]	0.00137**	-0.000334
	(0.000569)	(0.00177)
2nd Bed Tercile *(ref. 1st Tercile)*	-0.00117	0.00447
	(0.00164)	(0.00571)
3rd Bed Tercile *(ref. 1st Tercile)*	-0.00292*	0.00597
	(0.00140)	(0.00682)
Teaching	0.000628	-0.00532
	(0.00198)	(0.00349)
HHI [b]	-0.0189	0.00478
	(0.0109)	(0.0337)
Median Income (per $1,000s)	-0.00259*	-6.11e-05
	(0.00121)	(0.00505)
Uninsured %	0.00803	-0.00996
	(0.0311)	(0.0891)
Unemployment %	0.0105	0.291**
	(0.0163)	(0.0967)
Some College %	-0.00853	-0.00963
	(0.00720)	(0.0281)
Non-elderly with disability %	0.0111	-0.0230
	(0.0113)	(0.0461)
2018	0.00155***	0.00148
	(0.000413)	(0.00155)
2019	0.00307***	0.00264
	(0.000911)	(0.00176)
2020	0.00214*	-0.00304
	(0.00105)	(0.00258)
2021	0.000437	-0.0106**
	(0.00110)	(0.00359)
2022	0.000265	-0.0105**
	(0.00121)	(0.00375)
Constant	0.0385***	0.0314
	(0.00871)	(0.0268)

Abbreviations: DID, difference-in-differences; CI, confidence interval

© 2026 Santos T et al. *JAMA Network Open.*

The sample included all not-for-profit hospitals that had complete data from 2015 to 2022 (Oregon, n=42 or 336 hospital-years; and Control, n=540 or 4,320 hospital-years).

[a] Displays the coefficient from the difference-in-differences estimate using ordinary least squares regression adjusted for control variables (hospital bed size, teaching status, county median income, hospital market concentration as measured by the Herfindahl Hirschman Index, and the percent of the county population that was uninsured, unemployed, had some college education, and non-elderly with a disability). Models include hospital and calendar year fixed effects.

[b] In the hospital models, Herfindahl Hirschman Index was defined as the sum of all hospitals' squared market shares within each hospital referral region.

Standard errors clustered at the state level. *** p<0.01, ** p<0.05, * p<0.1

© 2026 Santos T et al. *JAMA Network Open.*

eTable 13. Anticipatory Effect Model: 2017-2018 Anticipatory Period vs. 2020-2022 Post-Policy Preemption Period; Full Estimates Output (Hospital) - Association of Oregon Financial Assistance Policy with Charity Care and Bad Debt, 2015-22

	**Charity Care**	**Bad Debt**
	n=210 hospital-years (Treatment)<br>n=2,700 hospital-years (Control)	
**DID (2019-2022 post)** [a]	0.000429	0.00229
	(0.000640)	(0.00259)
2nd Bed Tercile *(ref. 1st Tercile)*	-0.000499	-0.00143
	(0.00175)	(0.00515)
3rd Bed Tercile *(ref. 1st Tercile)*	-0.00293*	-0.000423
	(0.00145)	(0.00677)
Teaching	0.000563	-0.00382
	(0.00183)	(0.00343)
HHI [b]	-0.0221*	-0.00365
	(0.0115)	(0.0348)
Median Income (per $1,000s)	-0.00253*	-0.00127
	(0.00118)	(0.00643)
Uninsured %	0.00199	-0.0241
	(0.0341)	(0.109)
Unemployment %	0.0221	0.331**
	(0.0153)	(0.128)
Some College %	-0.00506	-0.0181
	(0.00631)	(0.0322)
Non-elderly with disability %	0.0136	-0.0554
	(0.0105)	(0.0513)
2018	0.00153***	0.00184
	(0.000441)	(0.00181)
2020	0.00217*	-0.00216
	(0.00104)	(0.00329)
2021	0.000462	-0.00967**
	(0.00108)	(0.00405)
2022	0.000281	-0.00941*
	(0.00120)	(0.00433)
Constant	0.0350***	0.0404
	(0.00805)	(0.0380)

Abbreviations: DID, difference-in-differences; CI, confidence interval

The sample included all not-for-profit hospitals that had complete data from 2015 to 2022 (Oregon, n=42 or 336 hospital-years; and Control, n=540 or 4,320 hospital-years).

© 2026 Santos T et al. *JAMA Network Open.*

[a] Displays the coefficient from the difference-in-differences estimate using ordinary least squares regression adjusted for control variables (hospital bed size, teaching status, county median income, hospital market concentration as measured by the Herfindahl Hirschman Index, and the percent of the county population that was uninsured, unemployed, had some college education, and non-elderly with a disability). Models include hospital and calendar year fixed effects.

[b] In the hospital models, Herfindahl Hirschman Index was defined as the sum of all hospitals' squared market shares within each hospital referral region.

Standard errors clustered at the state level. *** p<0.01, ** p<0.05, * p<0.1

© 2026 Santos T et al. *JAMA Network Open.*

eTable 14. Full Estimates Output (Hospital) - Association of Oregon Financial Assistance Policy with Charity Care and Bad Debt, 2015-22; Full Sample and By Metropolitan Status

	**FULL SAMPLE** [a]		**METROPOLITAN** [b]		**NON-METROPOLITAN** [c]	
	**Charity Care**	**Bad Debt**	**Charity Care**	**Bad Debt**	**Charity Care**	**Bad Debt**
	n=336 hospital-years (Treatment) n=4,320 hospital-years (Control)		n=208 hospital-years (Treatment) n=2,208 hospital-years (Control)		n=128 hospital-years (Treatment) n=2,112 hospital-years (Control)	
**DID (2019 post)** [d]	0.00612***	-0.00273	0.00599***	8.84e-06	0.0102***	-0.00507*
	(0.000866)	(0.00299)	(0.000828)	(0.00400)	(0.00142)	(0.00245)
**DID (2020 post)**	0.00451***	0.00216	0.00507***	0.00327	0.00145	0.00127
	(0.000776)	(0.00289)	(0.000822)	(0.00491)	(0.00121)	(0.00219)
**DID (2021 post)**	0.00277**	0.00813**	0.00258**	0.00996*	0.000935	0.00624*
	(0.000879)	(0.00335)	(0.00101)	(0.00504)	(0.000740)	(0.00328)
**DID (2022 post)**	-0.000492	0.00934**	-0.000532	0.0106**	-0.00294**	0.00784***
	(0.000962)	(0.00302)	(0.000997)	(0.00474)	(0.00103)	(0.00245)
2nd Bed Tercile *(ref. 1st Tercile)*	0.00171	-0.00417	-0.000401	0.00269	-0.000802	-0.00192
	(0.00219)	(0.00649)	(0.000566)	(0.00196)	(0.00290)	(0.0120)
3rd Bed Tercile *(ref. 1st Tercile)*	0.000157	0.000925	-0.00140	0.00680*	0.000694	-0.00406
	(0.00207)	(0.00502)	(0.00221)	(0.00355)	(0.00345)	(0.0113)
Teaching	-0.000486	-0.00439	-0.000359	-0.000330	0.00142	-0.0275***
	(0.00176)	(0.00275)	(0.00121)	(0.00396)	(0.00603)	(0.00782)
HHI [e]	-0.00938	0.00835	-0.00178	0.0484	-0.0124	-0.0355
	(0.00856)	(0.0195)	(0.0117)	(0.0275)	(0.00959)	(0.0316)
Median Income (per $10,000s)	-0.00110	-0.00108	0.00171	-0.0133**	-0.00184	0.00485
	(0.000916)	(0.00294)	(0.00125)	(0.00533)	(0.00131)	(0.00321)
Uninsured %	0.0391	0.181**	0.0705	0.264	0.0446*	0.175***
	(0.0244)	(0.0706)	(0.0430)	(0.184)	(0.0222)	(0.0484)
Unemployment %	-0.00234	0.0471	-0.0545	-0.111	-0.0128	0.102
	(0.0186)	(0.0750)	(0.0420)	(0.118)	(0.0306)	(0.0659)
Some College %	-0.00162	0.0256	-0.00432	0.0922	-0.000204	0.0122
	(0.00623)	(0.0267)	(0.0186)	(0.0619)	(0.00696)	(0.0205)
Non-elderly with disability %	0.0372**	-0.0956**	0.0608**	-0.172	0.0431	-0.0409
	(0.0141)	(0.0403)	(0.0222)	(0.121)	(0.0270)	(0.0488)
2016	0.00131	-0.000451	0.000508	0.00133	0.00197**	-0.000880
	(0.000830)	(0.00151)	(0.00120)	(0.00182)	(0.000746)	(0.00223)
2017	0.00318**	0.00440	0.000923	0.00671	0.00452***	0.00512
	(0.00106)	(0.00421)	(0.00103)	(0.00562)	(0.00119)	(0.00468)
2018	0.00453***	0.00672	0.00160	0.0121	0.00586***	0.00561
	(0.00122)	(0.00439)	(0.00141)	(0.00762)	(0.00125)	(0.00420)
2019	0.00551***	0.00771	0.00191	0.0167	0.00726***	0.00447
	(0.00156)	(0.00587)	(0.00193)	(0.00929)	(0.00171)	(0.00510)
2020	0.00433**	0.00154	0.000480	0.0135	0.00616***	-0.00237

© 2026 Santos T et al. *JAMA Network Open.*

	(0.00163)	(0.00494)	(0.00210)	(0.00822)	(0.00149)	(0.00450)
2021	0.00269	-0.00623	-0.00108	0.00458	0.00396**	-0.00980**
	(0.00169)	(0.00417)	(0.00212)	(0.00723)	(0.00154)	(0.00419)
2022	0.00262	-0.00632	-0.00122	0.00447	0.00401*	-0.00984**
	(0.00182)	(0.00378)	(0.00217)	(0.00695)	(0.00185)	(0.00392)
Constant	0.0145	0.0278*	-0.00544	0.0507	0.0118	-0.000834
	(0.00897)	(0.0148)	(0.0181)	(0.0330)	(0.00865)	(0.0252)

Abbreviations: DID, difference-in-differences; CI, confidence interval

Metropolitan defined as Rural Urban Continuum Code (RUCC) 1-3; Non-metropolitan defined as RUCC 4-9

[a] The sample included all not-for-profit hospitals that had complete data from 2015 to 2022 (Oregon, n=42 or 336 hospital-years; and Control, n=540 or 4,320 hospital-years).

[b] The sample limited to metropolitan counties included all not-for-profit hospitals that had complete data from 2015 to 2022 (Oregon, n=26 or 208 hospital-years; and Control, n=276 or 2,208 hospital-years).

[c] The sample limited to non-metropolitan counties included all not-for-profit hospitals that had complete data from 2015 to 2022 (Oregon, n=16 or 128 hospital-years; and Control, n=264 or 2,112 hospital-years).

[d] Displays the coefficient from the difference-in-differences estimate using ordinary least squares regression adjusted for control variables (hospital bed size, teaching status, county median income, hospital market concentration as measured by the Herfindahl Hirschman Index, and the percent of the county population that was uninsured, unemployed, had some college education, and non-elderly with a disability). Models include hospital and calendar year fixed effects.

[e] In the hospital models, Herfindahl Hirschman Index was defined as the sum of all hospitals' squared market shares within each hospital referral region.

Standard errors clustered at the state level. *** p<0.01, ** p<0.05, * p<0.1

eFigure 4. Event Study Analysis of Hospital Charity Care and Bad Debt Expenditures By Metropolitan Status, 2015-22

Charity Care as Percent of Operating Expenses: Non-Metropolitan [a]

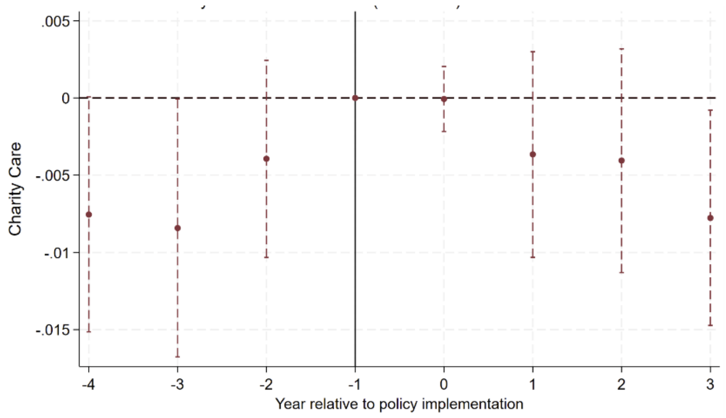

Charity Care as Percent of Operating Expenses: Metropolitan [b]

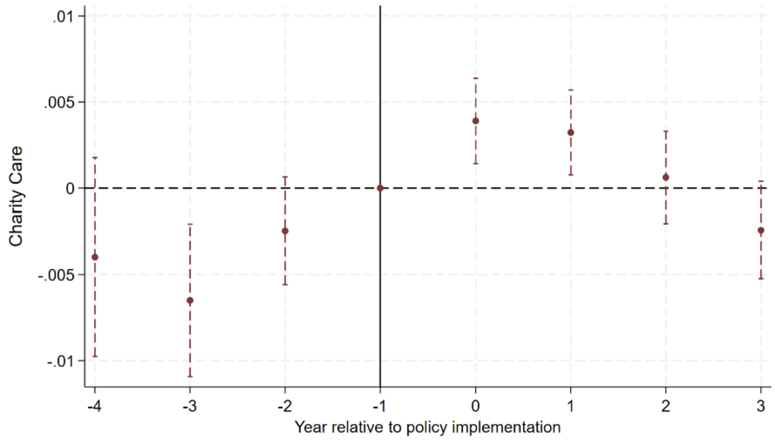

Metropolitan defined as Rural Urban Continuum Code (RUCC) 1-3; Non-metropolitan defined as RUCC 4-9

[a] The sample limited to non-metropolitan counties included all not-for-profit hospitals that had complete data from 2015 to 2022 (Oregon, n=16 or 128 hospital-years; and Control, n=264 or 2,112 hospital-years).

[b] The sample limited to metropolitan counties included all not-for-profit hospitals that had complete data from 2015 to 2022 (Oregon, n=26 or 208 hospital-years; and Control, n=276 or 2,208 hospital-years).

Models adjusted for control variables (hospital bed size, teaching status, county median income, hospital market concentration as measured by the Herfindahl Hirschman Index, and the percent of the county population that was uninsured, unemployed, had some college education, and non-elderly with a disability). Models include hospital and calendar year fixed effects.

95% CIs are calculated using standard errors clustered at the state level. Time at −1, the reference period, indicates the year before implementation of Oregon's financial assistance policy.

© 2026 Santos T et al. *JAMA Network Open.*

eFigure 4 (continued). Event Study Analysis of Hospital Charity Care and Bad Debt Expenditures By Metropolitan Status, 2015-22

Bad Debt as Percent of Operating Expenses: Non-Metropolitan [a]

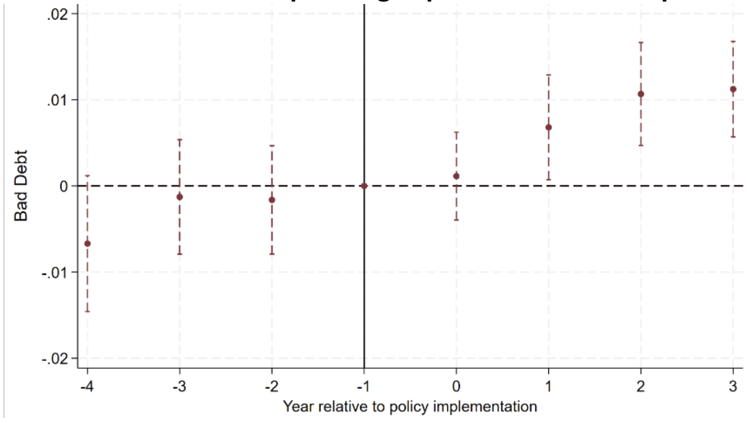

Bad Debt as Percent of Operating Expenses: Metropolitan [b]

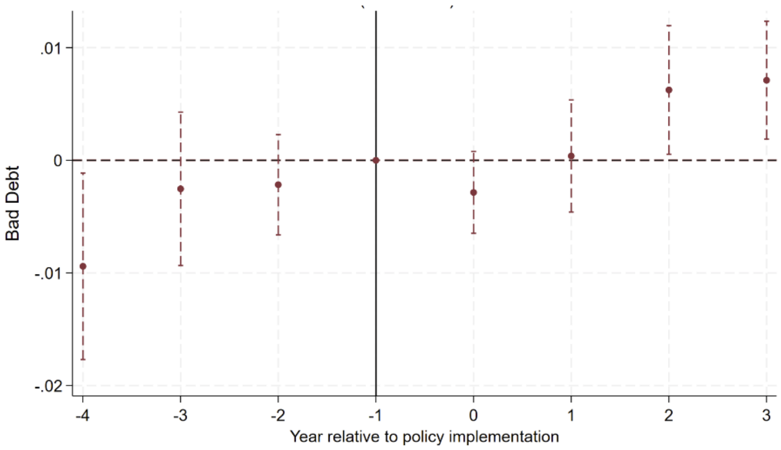

Metropolitan defined as Rural Urban Continuum Code (RUCC) 1-3; Non-metropolitan defined as RUCC 4-9

[a] The sample limited to non-metropolitan counties included all not-for-profit hospitals that had complete data from 2015 to 2022 (Oregon, n=16 or 128 hospital-years; and Control, n=264 or 2,112 hospital-years).

[b] The sample limited to metropolitan counties included all not-for-profit hospitals that had complete data from 2015 to 2022 (Oregon, n=26 or 208 hospital-years; and Control, n=276 or 2,208 hospital-years).

Models adjusted for control variables (hospital bed size, teaching status, county median income, hospital market concentration as measured by the Herfindahl Hirschman Index, and the percent of the county population that was uninsured, unemployed, had some college education, and non-elderly with a disability). Models include hospital and calendar year fixed effects.

95% CIs are calculated using standard errors clustered at the state level. Time at −1, the reference period, indicates the year before implementation of Oregon's financial assistance policy.

© 2026 Santos T et al. *JAMA Network Open.*

eFigure 5. Event Study Analysis of Hospital Charity Care and Bad Debt Expenditures By Tercile of Baseline Percent of Population =<400 FPL, 2015-22

Charity Care as a Percent of Operating Expenses

1st Tercile

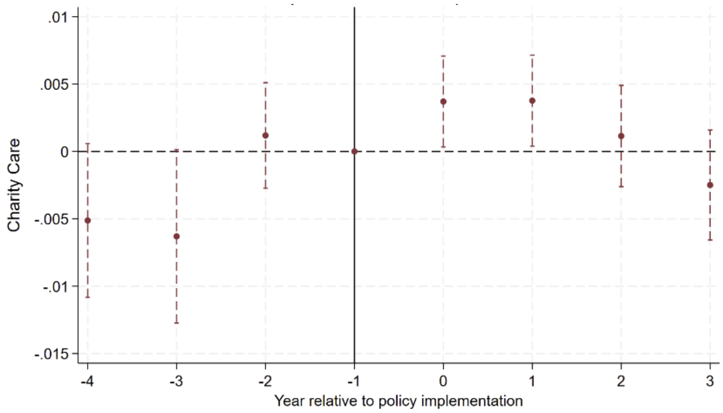

2nd Tercile

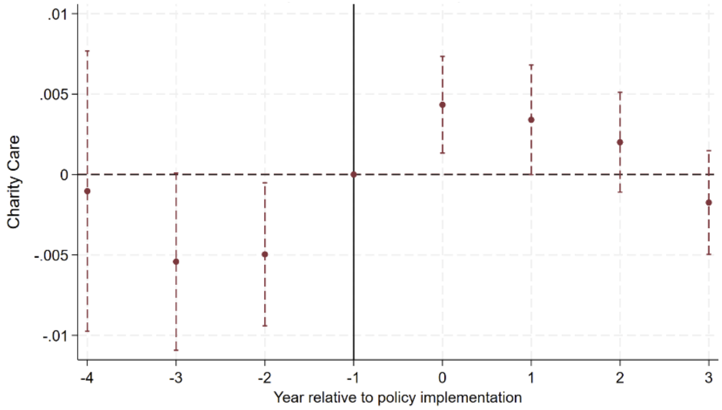

3rd Tercile

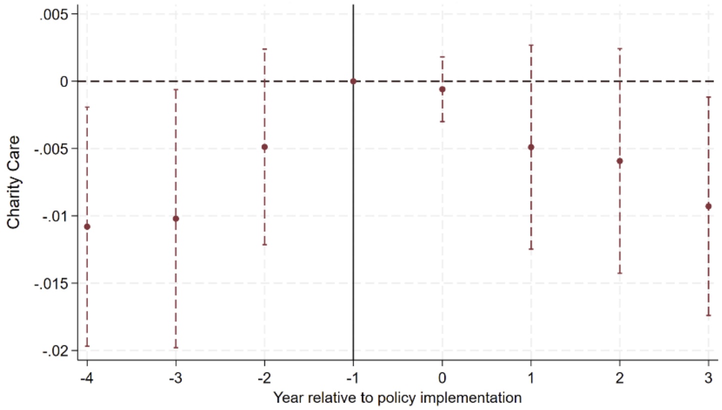

© 2026 Santos T et al. *JAMA Network Open.*

Each tercile included not-for-profit hospitals that had complete data from 2015 to 2022 (Oregon, n=14 or 112 hospital-years; and Control, n=180 or 1,440 hospital-years).

Models adjusted for control variables (hospital bed size, teaching status, county median income, hospital market concentration as measured by the Herfindahl Hirschman Index, and the percent of the county population that was uninsured, unemployed, had some college education, and non-elderly with a disability). Models include hospital and calendar year fixed effects.

95% CIs are calculated using standard errors clustered at the state level. Time at −1, the reference period, indicates the year before implementation of Oregon's financial assistance policy.

© 2026 Santos T et al. *JAMA Network Open.*

eFigure 5 (continued). Event Study Analysis of Hospital Charity Care and Bad Debt Expenditures By Tercile of Baseline Percent of Population =<400 FPL, 2015-22

Bad Debt as a Percent of Operating Expenses

1st Tercile

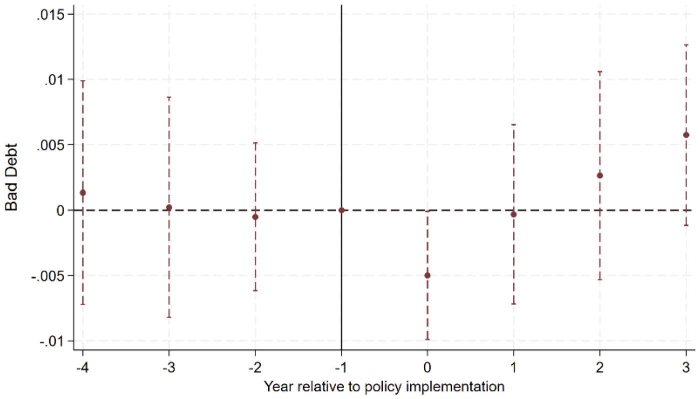

2nd Tercile

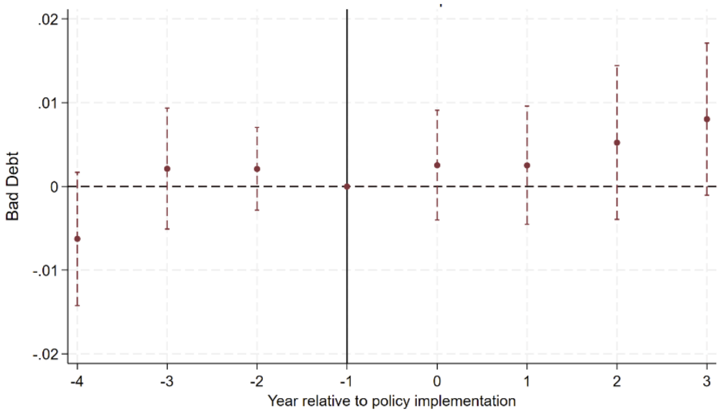

3rd Tercile

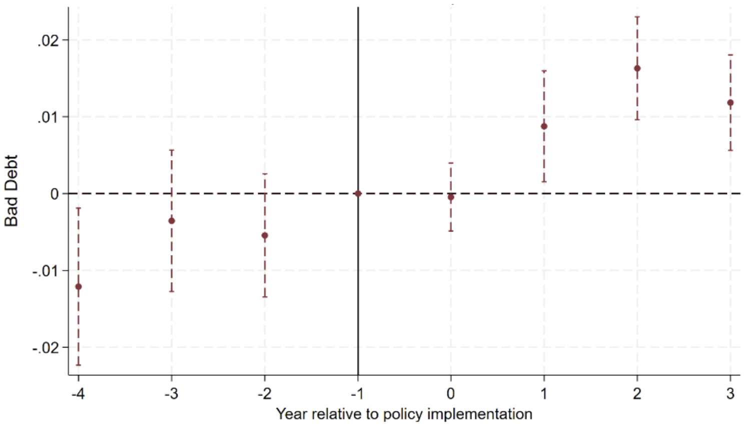

Each tercile included not-for-profit hospitals that had complete data from 2015 to 2022 (Oregon, n=14 or 112 hospital-years; and Control, n=180 or 1,440 hospital-years).

Models adjusted for control variables (hospital bed size, teaching status, county median income, hospital market concentration as measured by the Herfindahl Hirschman Index, and the percent of the county population that was uninsured, unemployed, had some college education, and non-elderly with a disability). Models include hospital and calendar year fixed effects.

95% CIs are calculated using standard errors clustered at the state level. Time at −1, the reference period, indicates the year before implementation of Oregon's financial assistance policy.

eFigure 6. Event Study Analysis of Hospital Charity Care for the Full Sample and By Metropolitan Status, 2015-22; Reference Period Set to 2015

Charity Care as a Percent of Operating Expenses – Full Sample [a]

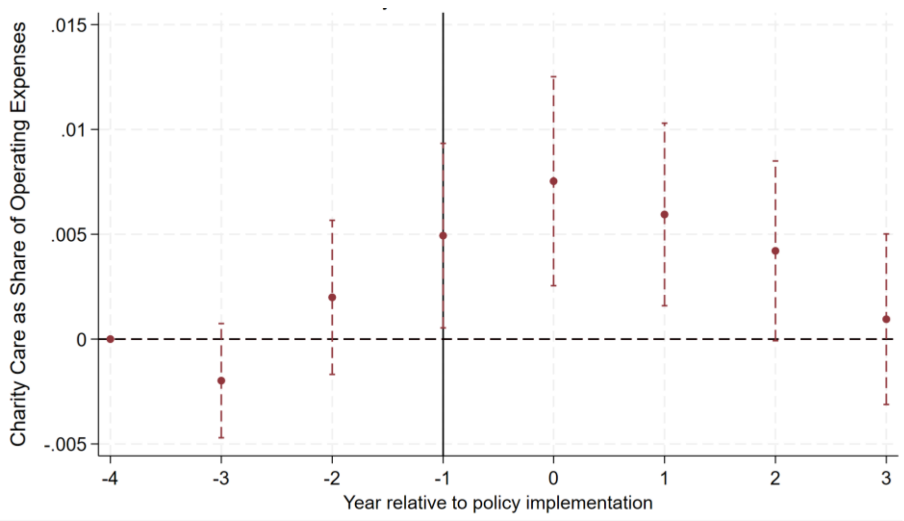

Charity Care as a Percent of Operating Expenses – Non-Metropolitan Hospitals [b]

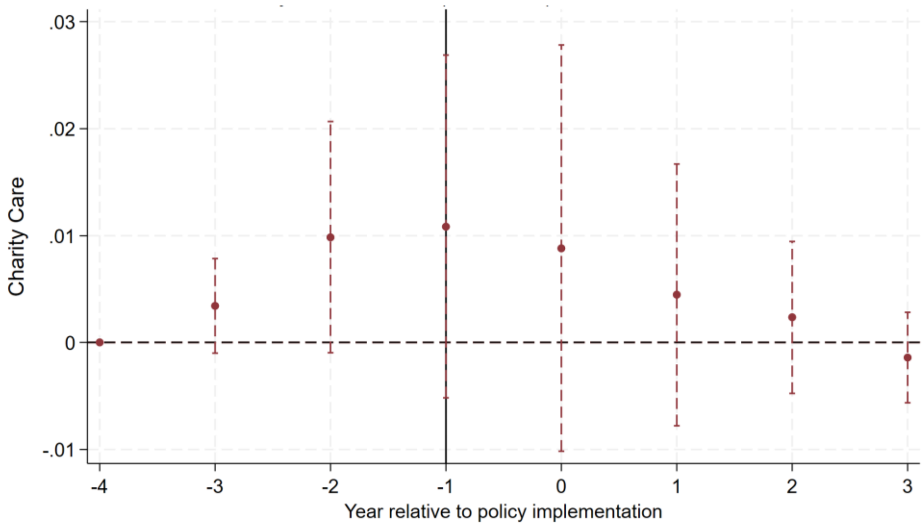

eFigure 6 (continued). Event Study Analysis of Hospital Charity Care for the Full Sample and By Metropolitan Status, 2015-22; Reference Period Set to 2015

Charity Care as a Percent of Operating Expenses – Metropolitan Hospitals [c]

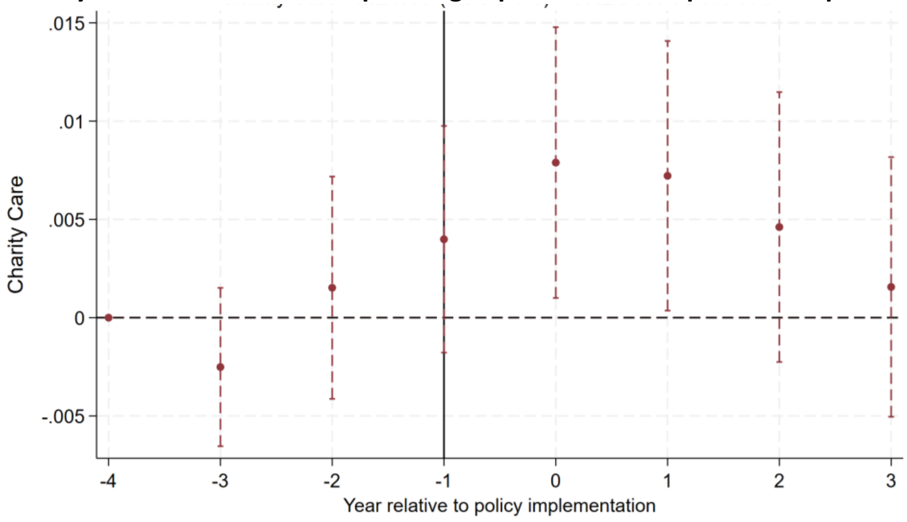

[a] The sample for the hospital analysis included all not-for-profit hospitals that had complete data from 2015 to 2022 (Oregon, n=42 or 336 hospital-years; and Control, n=540 or 4,320 hospital-years).

Metropolitan defined as Rural Urban Continuum Code (RUCC) 1-3; Non-metropolitan defined as RUCC 4-9

[b] The sample limited to non-metropolitan counties included all not-for-profit hospitals that had complete data from 2015 to 2022 (Oregon, n=16 or 128 hospital-years; and Control, n=264 or 2,112 hospital-years).

[c] The sample limited to metropolitan counties included all not-for-profit hospitals that had complete data from 2015 to 2022 (Oregon, n=26 or 208 hospital-years; and Control, n=276 or 2,208 hospital-years).

Models adjusted for control variables (hospital bed size, teaching status, county median income, hospital market concentration as measured by the Herfindahl Hirschman Index, and the percent of the county population that was uninsured, unemployed, had some college education, and non-elderly with a disability). Models include hospital and calendar year fixed effects.

95% CIs are calculated using standard errors clustered at the state level. Time at −4, the reference period, indicates the year 2015.

Note on Anticipatory Behavior by Oregon Hospitals:

It is possible that Oregon hospitals adjusted their financial assistance policies in anticipation of HB3076 being implemented. The study's first author (Dr. Santos) has maintained close contact with representatives from the Oregon Health Authority (OHA) who lead all efforts related to HB3076. Additionally, Dr. Santos has spoken to the original authors of the bill; that is, Service Employees International Union (SEIU) Local 49. SEIU representatives who were involved with drafting the language for HB3076 confirmed that the Oregon Association of Hospitals and Health Systems were key partners in these early efforts which began as early as 2018. Due to the hospital association's close involvement with drafting HB3076; it is reasonable to expect an anticipatory effect; that is, hospitals adjusted their

financial assistance policies ahead of HB3076's passage. This could be one explanation for the pre-trends we observed, specifically for the full sample (see Figure 2, Panel B in the paper) and for the metropolitan sample (see eFigure 4). Hospitals have full control in setting their financial assistance policies, whereas they don't have the same level of discretion in terms of bad debt costs (i.e., uncollectible hospital bills), and medical debt in collections. Furthermore, larger metropolitan hospitals may be better financially positioned to make changes to their financial assistance policies in anticipation of HB3076.

There were other legislative attempts to reform financial assistance policy in Oregon which predate HB3076. In 2017, HB2115 (see link below for details on the bill), a stricter regulation would have set a minimum community benefit standard of 5% of hospitals' gross receipts (i.e., charity care is part of community benefit). However, HB2115 did not make it out of committee. In early 2018, HB4084 (see link below for details on the bill) contained a lot of the same provisions as HB3076, and indeed, several provisions from HB4084 were worked into HB3076. Notably, we found several news articles (see links below) that suggest that the Oregon Association of Hospitals and Health System was closely monitoring and lobbying against the earlier efforts.

One way to empirically examine whether the anticipatory response by hospitals is driving the pre-trends is to set the reference period in the event study to be 2015 (i.e., the first year in the study period) and not 2018 which may be capturing some of the anticipatory effect.

Indeed, setting the reference period to −4 (2015; before policy negotiation began) eliminates the pre-trend, consistent with true parallel trends in the absence of anticipatory behavior. See eFigure 6 on pages 32-33 in the supplement.

6- **HB2115:** https://olis.oregonlegislature.gov/liz/2017R1/Downloads/MeasureDocument/HB2115/Introduced
7- **HB4084:** https://legiscan.com/OR/bill/HB4084/2018
8- **Media article about HB4084:** https://www.thelundreport.org/content/hospital-association-kills-greenlicks-attempt-clarify-charity-care-patients
9- **Media article on HB4084:** Oregon hospitals to clarify financial aid policies - KTVZ
10- **Media article on legislation for expanded financial assistance by hospitals:** https://www.thelundreport.org/content/oregon-legislators-could-limit-hospital-tax-breaks-increase-financial-scrutiny

eFigure 7. Robustness Check to COVID-19 Fiscal Shocks: Event Study Analysis Using Modified Total Margin as the Outcome

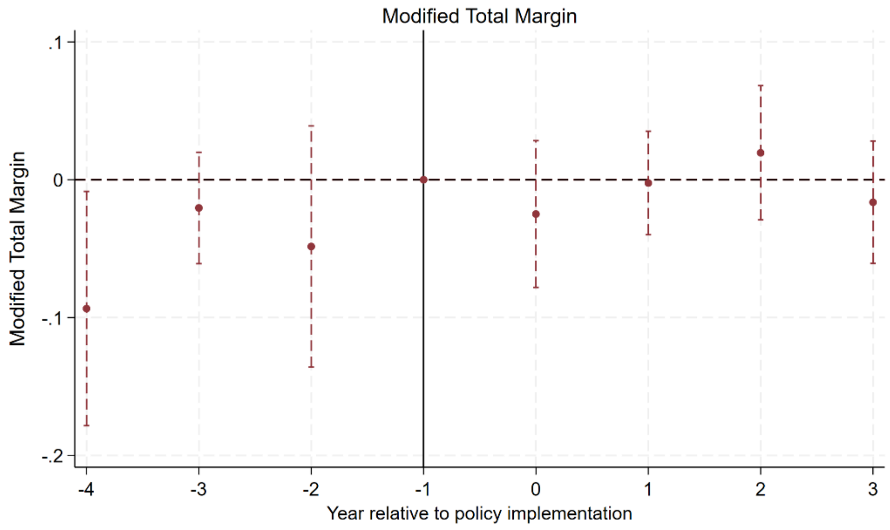

Note:
As a robustness check to COVID-19 fiscal shocks which could be a potential violation of the "common shocks" assumption of the difference-in-differences approach, we estimated a model using a modified total margin as the outcome. We calculated this modified total margin by subtracting all expenses reflected in the study's primary outcomes (i.e., charity care, bad debt, and operating expenses).

Specifications with modified total margins as the outcome tested whether the parallel trends assumption of difference in differences models was met over the entire sample period, including the period during the COVID-19 Public Health Emergency (PHE). This measure of profitability is an important determinant of charity care spending that takes into account overall financial performance during the PHE but excludes the financial impact of the policy by design. In this case, we'd expect the modified total margin to be unaffected by the Oregon FAP (i.e., parallel trends over the entire study period).

© 2026 Santos T et al. *JAMA Network Open.*

eTable 15. Robustness Check to COVID-19 Fiscal Shocks: Full Estimates Output - Association of Oregon Financial Assistance Policy with Charity Care and Bad Debt, 2015-22; By Tercile of Public Health Emergency Relief Funds

	**Charity Care**		
	**Terciles of Public Health Emergency Funds (2020-2022)**		
	**1st Tercile**	**2nd Tercile**	**3rd Tercile**
**DID (2019-2022)** [a]	0.00350***	0.00236**	0.00268*
	(0.00106)	(0.000962)	(0.00137)
2nd Bed Tercile *(ref. 1st Tercile)*	0.00551***	-0.000986	0.00274
	(0.000992)	(0.00290)	(0.00216)
3rd Bed Tercile *(ref. 1st Tercile)*	0.00285	0.000853	0.000658
	(0.00232)	(0.00297)	(0.00310)
Teaching	-0.000277	-0.00176	0.00335
	(0.00209)	(0.00243)	(0.00390)
HHI [b]	-0.0143	-0.0127	-0.0230
	(0.00874)	(0.0141)	(0.0188)
Median Income (per $1,000s)	-0.00179	-0.000339	-0.000300
	(0.00125)	(0.00143)	(0.00173)
Uninsured %	0.0560	0.0799	0.0307
	(0.0467)	(0.0599)	(0.0402)
Unemployment %	-0.0370	0.0296	0.0212
	(0.0377)	(0.0389)	(0.0200)
Some College %	-0.0124	0.00546	-0.00587
	(0.0119)	(0.0112)	(0.00831)
Non-elderly with disability %	0.0593	0.0484	0.0107
	(0.0405)	(0.0291)	(0.0140)
2016	0.000624	0.00227	0.00180**
	(0.00100)	(0.00144)	(0.000669)
2017	0.00366**	0.00402*	0.00282***
	(0.00148)	(0.00204)	(0.000839)
2018	0.00601***	0.00548*	0.00401***
	(0.00152)	(0.00279)	(0.00105)
2019	0.00783***	0.00673*	0.00441**
	(0.00184)	(0.00316)	(0.00163)
2020	0.00627**	0.00572*	0.00325*
	(0.00211)	(0.00315)	(0.00153)
2021	0.00338	0.00445	0.00215
	(0.00232)	(0.00319)	(0.00152)
2022	0.00298	0.00397	0.00228
	(0.00259)	(0.00348)	(0.00148)
Constant	0.0173	-0.000227	0.0176
	(0.0128)	(0.0184)	(0.0137)

© 2026 Santos T et al. *JAMA Network Open.*

Note:
The policy period overlaps with the COVID-19 pandemic, which may introduce confounding through changes in hospital demand and financial conditions.

This table reports difference-in-differences estimates stratified by terciles of Public Health Emergency (PHE) relief funds as a percentage of operating expenses (averaged across 2020–2022). This measure captures variation in hospitals' fiscal buffering during the pandemic. Positive and statistically significant effects appear across all terciles, indicating that pandemic-related financial shocks are unlikely to fully account for the estimated policy effect.

[a] Displays the coefficient from the difference-in-differences estimate using ordinary least squares regression adjusted for control variables (hospital bed size, teaching status, county median income, hospital market concentration as measured by the Herfindahl Hirschman Index, and the percent of the county population that was uninsured, unemployed, had some college education, and non-elderly with a disability). Models include hospital and calendar year fixed effects.

[b] In the hospital models, Herfindahl Hirschman Index was defined as the sum of all hospitals' squared market shares within each hospital referral region.

Models include county and calendar year fixed effects. Standard errors clustered at the state level. *** p<0.01, ** p<0.05, * p<0.1

eTable 15 (continued). Robustness Check to COVID-19 Fiscal Shocks: Full Estimates Output - Association of Oregon Financial Assistance Policy with Charity Care and Bad Debt, 2015-22; By Tercile of Public Health Emergency Relief Funds

	**Bad Debt**		
	**Terciles of Public Health Emergency Funds (2020-2022)**		
	**1st Tercile**	**2nd Tercile**	**3rd Tercile**
**DID (2019-2022)** [a]	0.00277	0.00225	0.000137
	(0.00372)	(0.00345)	(0.00183)
2nd Bed Tercile *(ref. 1st Tercile)*	-0.0262***	-0.000287	0.00757
	(0.00233)	(0.00154)	(0.00510)
3rd Bed Tercile *(ref. 1st Tercile)*	-0.0223***	0.00968	0.00726
	(0.00227)	(0.00539)	(0.00661)
Teaching	0.000887	-0.00189	-0.0128
	(0.00628)	(0.0109)	(0.0119)
HHI [b]	0.000444	-0.00175	0.0580
	(0.0252)	(0.0216)	(0.0512)
Median Income (per $1,000s)	-0.00647	-0.00421	0.00674
	(0.00574)	(0.00327)	(0.00423)
Uninsured %	-0.0116	0.162	0.197*
	(0.139)	(0.109)	(0.0889)
Unemployment %	0.252*	-0.103	0.0175
	(0.120)	(0.120)	(0.109)
Some College %	0.0237	0.0374	0.0343
	(0.0668)	(0.0411)	(0.0242)
Non-elderly with disability %	-0.0535	-0.137	-0.0581
	(0.0912)	(0.0842)	(0.0393)
2016	-0.000166	-0.00119	-0.00405
	(0.00164)	(0.00168)	(0.00288)
2017	0.00512	0.00147	0.00196
	(0.00403)	(0.00244)	(0.00704)
2018	0.00658	0.00497	0.00183
	(0.00537)	(0.00287)	(0.00684)
2019	0.00528	0.00876*	0.00115
	(0.00487)	(0.00471)	(0.00791)
2020	0.00216	0.00275	-0.00657
	(0.00548)	(0.00401)	(0.00686)
2021	-0.00369	-0.00393	-0.0164**
	(0.00527)	(0.00357)	(0.00624)
2022	-0.00375	-0.00398	-0.0161**
	(0.00528)	(0.00345)	(0.00593)
Constant	0.0219	0.0621*	-0.0163
	(0.0462)	(0.0324)	(0.0232)

© 2026 Santos T et al. *JAMA Network Open.*

Note:
The policy period overlaps with the COVID-19 pandemic, which may introduce confounding through changes in hospital demand and financial conditions.

This table reports difference-in-differences estimates stratified by terciles of Public Health Emergency (PHE) relief funds as a percentage of operating expenses (averaged across 2020–2022). This measure captures variation in hospitals' fiscal buffering during the pandemic. Positive and statistically significant effects appear across all terciles, indicating that pandemic-related financial shocks are unlikely to fully account for the estimated policy effect.

[a] Displays the coefficient from the difference-in-differences estimate using ordinary least squares regression adjusted for control variables (hospital bed size, teaching status, county median income, hospital market concentration as measured by the Herfindahl Hirschman Index, and the percent of the county population that was uninsured, unemployed, had some college education, and non-elderly with a disability). Models include hospital and calendar year fixed effects.

[b] In the hospital models, Herfindahl Hirschman Index was defined as the sum of all hospitals' squared market shares within each hospital referral region.

Models include county and calendar year fixed effects. Standard errors clustered at the state level. *** p<0.01, ** p<0.05, * p<0.1

© 2026 Santos T et al. *JAMA Network Open.*
