# Supplementary material for: Financial Assistance Policy, Hospital Charity Care, and Medical Debt in Collections
Source: JAMA Netw Open. 2026 Jan 27;9(1):e2555698. doi: 10.1001/jamanetworkopen.2025.55698 (PMC12848624; doi:10.1001/jamanetworkopen.2025.55698)
Supplement: Supplement 2. — Data Sharing Statement [file jamanetwopen-e2555698-s002.pdf]

Data Sharing Statement

Data
Data available: Yes
Data types: Other (please specify)
Additional Information: Data can be requested via an email to Tatiane Santos.
How to access data: Data can be requested via an email to Tatiane Santos
When available: With publication

Supporting Documents
Document types: None

Additional Information
Who can access the data: Researchers whose proposed use of the data has been approved
Types of analyses: For approved purposes.
Mechanisms of data availability: Without investigator support.
